# Addressing Stereotype Threat is Critical to Diversity and Inclusion in Organizational Psychology

**DOI:** 10.3389/fpsyg.2016.00008

**Published:** 2016-01-20

**Authors:** Bettina J. Casad, William J. Bryant

**Affiliations:** Department of Psychological Sciences, University of Missouri-St. Louis, St. LouisMO, USA

**Keywords:** stereotype threat, interventions, diversity, inclusion, workplace

## Abstract

Recently researchers have debated the relevance of stereotype threat to the workplace. Critics have argued that stereotype threat is not relevant in high stakes testing such as in personnel selection. We and others argue that stereotype threat is highly relevant in personnel selection, but our review focused on underexplored areas including effects of stereotype threat beyond test performance and the application of brief, low-cost interventions in the workplace. Relevant to the workplace, stereotype threat can reduce domain identification, job engagement, career aspirations, and receptivity to feedback. Stereotype threat has consequences in other relevant domains including leadership, entrepreneurship, negotiations, and competitiveness. Several institutional and individual level intervention strategies that have been field-tested and are easy to implement show promise for practitioners including: addressing environmental cues, valuing diversity, wise feedback, organizational mindsets, reattribution training, reframing the task, values-affirmation, utility-value, belonging, communal goal affordances, interdependent worldviews, and teaching about stereotype threat. This review integrates criticisms and evidence into one accessible source for practitioners and provides recommendations for implementing effective, low-cost interventions in the workplace.

Critics of stereotype threat research have four primary arguments: (1) mixed effects in operational high stakes testing environments (Cullen et al., 2004; Stricker and Ward, 2004; Sackett and Ryan, 2012); (2) necessary boundary conditions (Sackett, 2003; Sackett and Ryan, 2012; Ryan and Sackett, 2013); (3) lack of field studies (Kray and Shirako, 2012; Kalokerinos et al., 2014; Kenny and Briner, 2014; Streets and Major, 2014); and (4) impracticality of implementing workplace interventions (Streets and Major, 2014). Several publications have addressed the widely discussed arguments on high stakes testing (Cullen et al., 2004; Aronson and Dee, 2012; Sackett and Ryan, 2012; Walton et al., 2015a) and the boundary conditions of stereotype threat (Sackett, 2003; Sackett and Ryan, 2012; Ryan and Sackett, 2013). Throughout our review we provide evidence to counter the third and fourth criticisms on the lack of field studies and impracticality of workplace interventions.

## Overview

This review contributes to the growing attempt to apply research in the stereotype threat domain to the workplace (Aronson and Dee, 2012; Kang and Inzlicht, 2014; Walton et al., 2015a). We review the literature on the effects of stereotype threat beyond performance in an attempt to bring awareness to an area of stereotype threat research that may be underappreciated by practitioners due to its initial appearance as irrelevant (Kang and Inzlicht, 2014; Spencer et al., 2015). Highly relevant to I/O researchers and practitioners, stereotype threat can affect domain identification, job engagement, career aspirations, and openness to feedback. Another area that needs greater dissemination is the effects of stereotype threat in domains other than selection and high stakes testing, such as leadership, entrepreneurship, negotiations, and competitiveness. The content and organization of our review on the antecedents and consequences of stereotype threat in the workplace is similar to previous work (see Kray and Shirako, 2012; Kalokerinos et al., 2014). We complete the review by describing several institutional and individual level interventions that are brief, easily implementable, have been field tested, and are low-cost (summarized in **Table 1**). We provide recommendations for practitioners to consider how to implement the interventions in the workplace.

**Table 1:** Summary of stereotype threat interventions adaptable to the workplace.

Triggers of threat	Intervention	Psychological need(s) addressed	Supporting research
**Focus: institutional, structural**			
Stereotype-endorsing physical workplace environment (e.g., décor, location of restrooms)	Addressing environmental cues	Belonging	Elsbach, 2003; Ng and Burke, 2005; Braddy et al., 2006; Cheryan et al., 2009, 2011
Lack of personnel diversity, lack of same gender/race role models, color-blind diversity policies, ignorance of diversity issues	Valuing diversity	Belonging, being valued, group identity	Inzlicht and Ben-Zeev, 2000; Marx and Roman, 2002; Good et al., 2003; Murphy et al., 2007; Plaut et al., 2009; von Hippel et al., 2011a
Entity views of intelligence, cross-race and gender critical feedback	Wise feedback	Competence, trust	Cohen et al., 1999; Yeager et al., 2013
Entity views of intelligence	Organizational mindset	Competence	Dweck, 1999, 2006; Murphy and Dweck, 2010; Emerson and Murphy, 2015
**Focus: individual, psychological**			
Diagnostic tasks, stable, internal, uncontrollable attributions for failure	Reattribution training	Competence, belonging	Wilson et al., 2002; Good et al., 2003; Roberson and Kulik, 2007; Walton and Cohen, 2007; Jamieson et al., 2010
Diagnostic tasks	Reframing the task	Competence, Attenuation of task-related anxieties	Spencer et al., 1999; Quinn and Spencer, 2001
Domain-relevant trait assessment or diagnostic tasks	Values-affirmation	Self-integrity, self-identity, social worth, competence	Cohen et al., 2006, 2009; Martens et al., 2006; Sherman and Cohen, 2006; Rydell et al., 2009; Sherman et al., 2013; Walton et al., 2015a
Diagnostic tasks, low task interest and motivation	Utility-value	Competence, identity, sense of purpose	Harackiewicz et al., 2008, 2015; Hulleman et al., 2008; Brown et al., 2015; Smith et al., 2015
Numeric underrepresentation, stereotypic environmental cues	Belonging	Belonging, self-worth, being valued	Walton and Cohen, 2007, 2011; Walton et al., 2015b
Emphasis on agentic goals, independent worldviews, cultural mismatch	Communal goal affordances, interdependent worldview	Person-environment fit, values, identity, consistency, congruence	Diekman et al., 2010; Stephens et al., 2012a,b; Smith et al., 2014, 2015; Thoman et al., 2015
Any trigger of stereotype threat	Teaching about stereotype threat	Belonging, competence, self-worth, group identity	Kray et al., 2001, 2004; Johns et al., 2005, 2008; Gupta et al., 2008


## Effects of Stereotype Threat Beyond Performance

When research on stereotype threat was first published, the focus was on academic test performance for women and racial minorities (Steele and Aronson, 1995). However, since this time research has expounded, cataloging numerous psychological, and behavioral outcomes that are affected by experiencing stereotype threat (Schmader et al., 2008; Inzlicht et al., 2012). Research on *stereotype threat spillover* has documented pernicious effects of stereotype threat beyond performance (Inzlicht and Kang, 2010; Inzlicht et al., 2011). Research on stereotype threat in an I/O context similarly has focused on performance as the key outcome (e.g., Sackett et al., 2001; Sackett and Ryan, 2012). It seems that because the effects of stereotype threat in high-stakes testing has been controversial (Kalokerinos et al., 2014), the overemphasis on performance may have undermined I/O psychology’s research focused on other outcomes (Kray and Shirako, 2012; Kang and Inzlicht, 2014). Indeed, research demonstrates that stereotype threat spillover effects are likely *underestimated* and may account for some of the null findings of stereotype threat on performance in field studies (Inzlicht and Kang, 2010; Inzlicht et al., 2011; Kang and Inzlicht, 2014). In this section, we first describe the psychological processes responsible for stereotype threat spillover effects. We then review research showing that stereotype threat negatively impacts outcomes beyond performance (see Spencer et al., 2015). These negative outcomes are critical for I/O practitioners to consider when evaluating the usefulness of stereotype threat in the workplace. Although there are many outcomes affected by stereotype threat including intrapersonal, interpersonal, and employer–employee outcomes (Kray and Shirako, 2012; Kalokerinos et al., 2014), we focus on four outcomes that are linked to other downstream effects relevant to the workplace: openness to feedback (Roberson et al., 2003), domain identification (Crocker et al., 1998), job engagement (Harter et al., 2002), and reduced career aspirations (Davies et al., 2005).

### Stereotype Threat Processes

After many studies established the effects of stereotype threats on various outcomes for several minority groups, research turned to understanding the mechanisms driving these effects (Schmader et al., 2008; Inzlicht et al., 2014). Experiencing stereotype threat can lead to a cascade of processes that include attentional, physiological, cognitive, affective, and motivational mechanisms (see Casad and Merritt, 2014). When a stigmatized person becomes aware that their stigmatized status may be relevant in a particular context, they may become vigilant and increase attention for environmental cues relevant to potential prejudice and discrimination.

In addition to increased vigilance or attention, stereotype threat causes heightened physiological arousal such as heighted blood pressure and vasoconstriction (Blascovich et al., 2001; Croizet et al., 2004; Murphy et al., 2007; Vick et al., 2008). However, physiological arousal alone does not necessarily lead to negative outcomes, but rather the appraisal of a stimulus as threatening or challenging elicits a response (Blascovich et al., 2004a,b; Schmader et al., 2008; Inzlicht et al., 2012).

Research on stereotype threat processes has identified cognitive and affective factors, particularly cognitive, and affective appraisals, as determinants of outcomes (Major et al., 2002; Major and O’Brien, 2005). Cognitive appraisals can heighten awareness of a relevant stereotype, thus reinforcing the arousal of threat (Inzlicht et al., 2006a). These cognitions include the extent to which a stressor is self-relevant, dangerous, and creates uncertainty. The negative cognitions initiate physiological arousal, such as elevated cortisol, increased adrenaline, increased blood pressure, and other cardiovascular responses such as increased vasoconstriction (Chen and Matthews, 2003; Blascovich et al., 2004a; Vick et al., 2008). Relatedly, affective appraisals can heighten awareness of a relevant stereotype, thus reinforcing the arousal of threat (Inzlicht et al., 2006a). These emotions include feeling overwhelmed, nervous, anxious, worried, and fearful, which initiate physiological arousal like cognitive appraisals (Chen and Matthews, 2003; Blascovich et al., 2004a).

A final mechanism that explains why stereotype threat can negatively affect performance and spill over into other domains is executive functions. Executive functions are required to self-regulate one’s thoughts, feelings, and behaviors under stress (Muraven et al., 1998; Muraven and Baumeister, 2000). This self-regulation requires not only motivation, but also ego-strength, which comes in limited supplies (Muraven et al., 1998; Muraven and Baumeister, 2000). When a task requires a controlled response, willful action can quickly deplete ego-strength, or it can divert motivation and attention to other actions (Inzlicht et al., 2014). Research has shown that women under stereotype threat were quicker to fail at a self-regulation task (squeezing a hand grip—a task irrelevant to math-based stereotype threat) than women not under threat (Inzlicht et al., 2006b). Other research shows that participants under threat give up on complex tasks more quickly than participants not under threat (Inzlicht and Hickman, 2005, Unpublished Manuscript). In order to overcome stereotype threat, people have to exert self-control, often having to work harder to maintain performance in the face of threat (Inzlicht and Kang, 2010). Exerting self-control may prevent negative performance at the moment, possibly accounting for null effects of stereotype threat on performance in workplace settings; however, exerting self-control comes at a cost. The stress of working against stereotype threat can spill over into other seemingly unrelated domains such as health (diet, exercise, and alcohol/drug abuse), decision-making, and aggression (Inzlicht and Kang, 2010; Inzlicht et al., 2011). Next, we describe four negative consequences of stereotype threat beyond performance.

### Reduced Openness to Feedback

Stereotype threat has been shown to hinder affected employees’ openness to and utilization of critical feedback (Roberson et al., 2003). Feedback is vital for an organization’s workforce to adapt and grow, and when employees from stigmatized groups are not able to utilize feedback as effectively as non-stigmatized workers, their chances for advancement and success will be hindered (Crocker et al., 1991).

Employees faced with stereotype threat often find it easy to assume that their coworkers or superiors are biased against them due to their group membership (Walton et al., 2015a). This can often occur when a non-minority manager presents negative, though constructive, feedback to a minority subordinate. If the employee is vulnerable to stereotype threat, such as being a numeric minority in the workgroup, they are more likely to interpret negative feedback as internally attributed, such that it speaks to their inherent ability (Kiefer and Shih, 2006). This misattribution increases the vulnerability of self-esteem, so these employees may then be more likely to interpret that negative feedback as biased and discount it (Roberson et al., 2003). Discounting valuable feedback robs the employee of a valuable learning experience and the opportunity to improve their standing or performance (Roberson et al., 2003). A non-minority employee does not undergo this process when interpreting feedback, so they can more easily perceive the feedback as legitimate and utilize it effectively.

The tendency to discount critical feedback has been documented in several studies. Cohen et al. (1999) found that African American students were less likely to adjust written essays that following feedback given by white professors if they were led to believe that white students received less negative feedback. Cohen and Steele (2002) found a similar effect with female science students when giving presentations before and after negative feedback. It is likely that this pattern is due to minority members’ desire to protect their self-esteem from negative information regarding personal performance. Because subtle forms of prejudice are pervasive, it is highly likely for stereotyped individuals to assume that feedback in interracial or mixed gender context might be biased. Therefore, discounting negative feedback to protect one’s self-esteem may be adaptive, reasonable, and justified. Failing to discount biased feedback could potentially reinforce negative stereotypes about belonging and ability (Crocker and Major, 1989; Cohen et al., 1999; Walton et al., 2015a).

Apart from discounting feedback from supervisors, stereotype threat may influence how minority employees seek out feedback concerning their performance. Research has shown that direct feedback, or explicit and outright feedback, is much more effective in terms of improving performance. Conversely, indirect feedback, or monitoring one’s environment for cues about ones performance, is much more ambiguous and therefore less useful (Ashford and Tsui, 1991). An important distinction, however, is that direct feedback can often be perceived as emotionally threatening as it reflects a more true representation of performance. Indirect feedback is much less threatening because the recipient is not confronted about their performance outright (Ashford and Northcraft, 1992). In order to protect social standing and avoid public scrutiny, minority employees may actively avoid direct feedback (Roberson et al., 2003).

### Reduced Domain Identification

Chronic exposure to threat may lead stigmatized individuals to disidentify from the domain in which they are negatively stereotyped (Steele and Aronson, 1995). Disidentification serves as a coping mechanism to chronic threat where individuals selectively disengage their self-esteem from intellectual tasks or domains (Steele, 1992, 1997; Crocker et al., 1998). That is, by redefining their self-concept to not include achievement in that domain as a basis for self-evaluation, individuals protect their self-esteem so that poor performance in that domain is no longer relevant to their self-evaluation. However, disidentification is a maladaptive response, and it is a contributing factor to reduced career and performance goals (Major and Schmader, 1998) and workplace turnover (Crocker et al., 1998; Harter et al., 2002).

Another area of concern is that stereotype threat interferes with minorities’ ability to integrate personal identities with professional identities. When employees view their personal identity (e.g., woman, African American) as incompatible with their professional identity (e.g., lawyer) because of stereotype threat in the workplace, negative mental health consequences are likely (Settles et al., 2002; Settles, 2004). Female lawyers, accountants, and managers who experienced stereotype threat reported separating their identity as a woman from their professional identity (von Hippel et al., 2010, 2011a, 2015). Other research shows that women scientists report having to switch back and forth between their identity as a woman and identity as a scientist in order to fit into male-dominated environments (Settles, 2004). Adverse consequences of this lack of identity integration include negative job attitudes (von Hippel et al., 2011a), more negative work-related mental health (von Hippel et al., 2015) greater depression (Settles, 2004), lower life satisfaction (Settles, 2004), and reduced likelihood of recommending fellow women to the field (e.g., finance; von Hippel et al., 2015).

### Reduced Engagement

Another non-performance consequence of stereotype threat is the tendency for stereotyped individuals to disengage from their work tasks and the feedback that follows. Employees under threat may disengage in order to distance their self-esteem from the potential consequences of their work performance (Major and Schmader, 1998). If a particular stereotype indicates that the individual will perform poorly, that individual is more likely to reduce their attachment to their performance for fear of potentially proving that stereotype correct. This process leads to feelings of powerlessness (Major et al., 1998). Stigmatized individuals therefore reduce the amount of care and concern they put toward a work outcome in order to avoid the negative consequences of their anticipated poor performance. Individuals who identify highly with their domain are most susceptible to disengagement, since success in that domain is more central to them, making negative feedback much more damaging.

Disengagement is closely related to disidentification in that repeated disengagements often contribute to the individual reducing their identification with a certain domain. Disengagement is typically a state-level phenomenon that occurs in response to specific situations, such as analyzing scientific data, whereas disidentification is typically a chronic state that affects the individual’s overall identity attachment to the domain, such as being a scientist. If the individual regularly disengages from relevant tasks in order to shield his or her self-esteem, a reduction of identification to the domain could result. This cycle is problematic, as it indicates disengagement can ultimately result in higher turnover due to a lack of domain identification (Crocker et al., 1998; Harter et al., 2002).

Disengagement has been shown to negatively impact task performance and motivation, such that individuals will give up more easily on a stereotype-relevant task while under threat (Crocker and Major, 1989; Steele, 1992; Major and Schmader, 1998). Research indicates it is not the task itself that is threatening, but rather the anticipated feedback that follows (Ashford and Tsui, 1991). If employees under threat are highly engaged in their work, and they receive negative feedback that aligns with a relevant group stereotype, it could be much more damaging to their self-esteem than it would be for non-threatened employees (Major and Schmader, 1998).

Disengagement results from discounting and devaluing. Discounting occurs when the employee dismisses feedback as an invalid representation of one’s potential due to external inadequacies, such as skepticism toward an intelligence test. Devaluing occurs when the employee dismisses the importance of the feedback, often taking the position that the feedback does not matter to them or their career path. When stereotyped individuals engage in discounting and devaluing, negative feedback is less likely to affect self-esteem because the feedback has been deemed irrelevant or flawed (Major and Schmader, 1998).

Interestingly, there has been a small body of research investigating the potential adaptiveness of disengagement. For example, Nussbaum and Steele (2007) observed that temporarily disengaging from harmful feedback can actually foster persistence, as it deflects damage to the self-esteem which would otherwise create a sense of lack of belonging. While it is possible that situational disengagement could be beneficial in particular contexts, it cannot be harnessed and applied to particular contents of the individual’s choosing – it is evoked whenever the individual feels threatened. Additionally, disengagement, regardless of its capacity to protect self-esteem, results in the rejection of valuable feedback that could otherwise be used toward refining work-relevant skills. Finally, chronic disengagement has been shown to lead to disidentification, or no longer perceiving one’s workplace identity as central to self-identity (Crocker et al., 1998), which in turn is associated with increased turnover (Harter et al., 2002). It is therefore critical that disengagement is curtailed, and reducing stereotype threat is necessary to do so.

### Reduced or Changed Career Aspirations

Another consequence of chronic experiences with stereotype threat is reduced or altered career aspirations. When people feel threat in a domain, they often feel they have fewer opportunities for success in the domain (Steele, 1997). For example, Davies et al. (2005) found that women were less interested in taking on leadership roles after viewing gender stereotypic television commercials. Similarly, when leadership roles are described using masculine traits, women report less interest in entrepreneurship than men (Gupta et al., 2008). Reduced career aspirations in response to threat, particularly for women in leadership, entrepreneurship, and science may exacerbate the gender gap in these fields (Murphy et al., 2007; Koenig et al., 2011).

## Consequences of Stereotype Threat for Organizations

As previously outlined, stereotype threat leads to a cascade of mechanisms that can lead to poor performance in a stereotyped domain, or spillover into unrelated domains such as health. In the previous section we described research documenting how stereotype threat can result in reduced openness to feedback from employers, reduced domain identification, reduced job engagement, and reduced or altered career aspirations. All four of these consequences are linked to changes in behaviors that have consequences for the workplace. Experiencing stereotype threat has shown to impair leadership performance and aspirations, negotiation skills, entrepreneurial interests, and skills, and desire to work in competitive environments and competiveness skills (Kray and Shirako, 2012). The following section describes predominantly lab-based research that shows the negative effects of stereotype threat on these four important workplace behaviors.

### Leadership

Encountering stereotype threat has been shown to limit one’s willingness to embrace challenges and work through uncertainty because any resulting failure could be interpreted as evidence supporting the stereotype (Steele, 1997). Experiencing stereotype threat leads individuals to avoid domains in which they are stereotyped as not belonging, such as women in leadership. Leaders are commonly assumed to be white males (Koenig et al., 2011), therefore women and racial minorities seeking leadership positions must directly challenge that stereotype. Empirical evidence has supported the idea that when individuals face stereotype threat, they are less likely to pursue leadership roles, particularly when they are the only member of their group among their peers (Hoyt et al., 2010). It is assumed that the threatening environment activates a heightened aversion to risk, which when coupled with greater uncertainty regarding their success, may cause them to forgo challenges such as striving for leadership roles.

Aligning with this theory, Davies et al. (2005) instructed women to choose to hold either a leadership or non-leadership position following the presentation of either a stereotype-activating commercial or a neutral commercial. Results indicated that women who viewed the stereotype-relevant commercial were more likely to elect to hold the non-leadership position, whereas those who viewed the neutral commercial were more evenly distributed between the two roles. This indicates that the knowledge and activation of stereotypes of women’s roles as subordinate or supportive in nature rather than leadership roles will diminish women’s desire to lead due to the fear of confirming the stereotype. This phenomenon is even more dangerous because it can activate a self-perpetuating cycle – stereotyped individuals avoid leadership roles due the stereotype that leaders should be white males, which then discourages those individuals to establish a prominent leadership presence. When no female or minority leaders are present, no information counter to the stereotype is available and the stereotype persists.

It is important to note that individual differences can diminish the effects of stereotype threat on leadership aspirations. For example, for women who are already high in leadership self-efficacy, the presence of stereotypes can actually motivate them to pursue leadership positions and increase their identity as a leader (Hoyt, 2005). Research has shown that identity safety can mitigate the effects of stereotype threat (Markus et al., 2000), meaning security with one’s identity can increase a feeling of belonging in that particular domain. Stereotyped individuals can therefore view the stereotype as a challenge rather than a threat and feel less uncertainty regarding future success. One issue, however, is that establishing leadership self-efficacy often requires past performances that were successful (Bandura, 1977), which means that in order for leadership self-efficacy to be high enough for stereotyped individuals to challenge stereotypes, it may be necessary for them to have proven their capability as a leader at an earlier time.

### Entrepreneurship

Paralleling the reduced aspiration to participate in leadership roles, the presence of stereotype threat can also inhibit individuals from pursuing entrepreneurial endeavors. Many traits that are important for leaders are also important for entrepreneurial success (e.g., assertiveness, risk-taking), thus similar hesitations can result. Although it appears that the number of female entrepreneurs is growing in industries such as retail and personal service (Anna et al., 2000), this is presumably because those industries still center on female-oriented traits such as nurturance, sensitivity, and fashion-sense. Even with this increase, however, the number of male entrepreneurs still outnumbers that of female entrepreneurs 2 to 1 (Acs et al., 2005).

When stereotype threat is due to contextual or situational cues, individuals can strive to eliminate the threat by distancing themselves from that situation or context. Because masculine stereotypes are important for entrepreneurial success, women may negatively evaluate their capability for success and therefore distance themselves from any entrepreneurial endeavor. Although some research has shown that proactive personalities can buffer the effect of stereotype threat on women’s entrepreneurial intentions (Gupta and Bhawe, 2007), activating stereotypes of entrepreneurship and masculinity discourages women from taking such risks.

### Negotiations

Many of the stereotypic masculine traits mentioned previously can impact aspects of the workplace other than career aspirations and risk-taking. Because strong negotiators are stereotyped to have masculine qualities, women may alter their negotiation strategies. Much of this research is similar to other areas, namely that activating gender stereotypes can cause women to underperform during negotiations compared to when stereotypes are not activated (Kray et al., 2002). Stereotype threat also leads to less willingness to initiate a discussion that is negotiative in nature Small et al. (2007).

The dynamic nature of negotiations makes it challenging for researchers to determine whether gender differences in negotiation performance are due to the suppressed performance of women under threat, or the situational control experienced by male opponents (Kray and Shirako, 2012). Although research suggests the mere competitive nature of the negotiation process is what deters women from pursuing maximum benefits (Gneezy et al., 2003; Niederle and Vesterlund, 2010), several studies show women’s negotiation performance improves when stereotypes are made explicit (Kray et al., 2001, 2004). This phenomenon is due to stereotype reactance, or the tendency to react counter to a stereotype when overt attention is drawn to its unfairness. However, if a stereotype is presented implicitly, women’s performance may still be negatively impacted (Kray et al., 2001).

The negative effects of stereotype threat on negotiations does not necessarily stop at the bargaining table. Research has shown that women who behave in ways counter to gender stereotypes may be faced with social backlash (von Hippel et al., 2011b), especially if interactions with the negotiator are expected to recur. This suggests that even if women are able to overcome stereotype threat and negotiate effectively in a particular situation, the chronic experience of stereotype threat can potential impact women throughout their careers.

### Competitiveness

As previously mentioned, one reason women may be less effective in leadership, entrepreneur intentions, and negotiations is a dislike of competitiveness. Competitive environments can be threatening to women due to the stereotype that women cannot fend for themselves when competing with men, and that they are better suited for supportive roles. Gneezy et al. (2003) conducted an experiment where participants were instructed to complete a computerized maze to earn compensation. Participants were either compensated for every maze completed regardless of performance or only if they solved the most puzzles in a set amount of time. Results indicated that men’s and women’s performances did not differ in the non-competitive condition, but women’s performance was significantly lower in the competitive condition. In the competitive condition, women elected not to dedicate effort to compete due to a preconceived expectation of losing. This parallels the idea that women may not feel capable of performing well in competitive environments, and therefore do not fully engage themselves, which can protect their self-esteem following expected loss (Gneezy et al., 2003).

People who lack a competitive nature may experience difficulties in the competitive world of work. Stereotypes that give men a competitive edge (e.g., men play sports while women cheer them on) can carry over into a wide array of workplace contexts, potentially leaving women feeling unprepared, or incapable of competing. Although some research shows that women are capable of competitiveness on tasks in which women are more knowledgeable (Günther et al., 2010), this stereotypical gender difference still gives a normative advantage to males across most situations. In a workplace, whether it be for a position, client, project, or ethical dilemma, having the ability and motivation to compete with others may determine success or failure. Understanding this phenomenon and equipping women with strategies to be competitive in the workplace is of vital importance.

## Stereotype Threat Interventions in the Workplace

Broadly speaking, stereotype threat research is typically divided into three subdivisions – whether stereotype threat is present in a given domain, whether its presumable effects can be prevented or reduced, and the underlying mechanisms of the effects. All three types of research are necessary – there is no use preventing it if its effects are nonexistent, but no change will ever occur if we do not first understand why it is happening and then develop strategies to overcome it. Research has come a long way in developing intervention strategies, and there now exists a wide variety of interventions that organizations can implement in order to reduce stereotype threat and its effects on employees.

One issue concerning these interventions raised by researchers and organizational leaders is that many of the strategies, while sound in theory and laboratory testing, are not always applicable or practical in real-world practice, and therefore are not helpful to organizations (Streets and Major, 2014). For example, one well-known intervention strategy within the stereotype threat literature is to increase minority representation within the organization (Purdie-Vaughns et al., 2008; Spencer et al., 2015). Doing so has been shown to not only increase the value placed on diversity, but has also aided in the development of role models—a strong antecedent for the success of stereotyped individuals (Marx and Roman, 2002). While this practice is undoubtedly effective, reorganizing personnel or modifying hiring practices requires major organizational change and expense. This intervention may not be attainable, particularly for smaller companies with fewer resources and opportunities to hire new personnel.

It seems the main argument against implementing stereotype threat interventions in the workplace is cost and potential disruption to the work environment. In the next section we describe intervention strategies that are no or low cost that can be integrated into existing training programs. Ultimately the organization has to weigh the costs and benefits of implementing workplace interventions. However, continuing to ignore diversity issues in the workplace and having employees who experience stereotype threat may negatively impact organizations’ bottom line in unanticipated ways (e.g., higher turnover, burnout, lawsuits).

Stereotype threat is triggered by subjective interpretation of situational contexts, which makes perceptions malleable through interventions. Interventions target institutional, structural level features of the organization and also individual level factors related to subjective construals of environments (Cohen et al., 2012). Effective interventions range from brief, low-cost interventions such as changing physical workplace environments to long-term, high-cost changes such as diversifying the workforce. In this section we describe a range of stereotype threat reducing interventions that have been tested in laboratory and field settings, which are summarized in **Table 1.**

### Institutional and Structural Level Interventions

#### Addressing Environmental Cues

Research has documented several environmental cues that can trigger stereotype threat, thus employers can be proactive in minimizing the presence of these cues in the workplace. Regarding the physical workplace environment, décor can signal to employees, and prospective recruits, whether they are welcomed in the organization. For example, halls decorated with photos of senior management and executives that represent Caucasian males may trigger doubt that women and minorities can advance in the organization. Other seemingly benign objects, such as the choice of magazines in a reception area, can affect the perception of the organization’s diversity values (Cohen and Garcia, 2008). Do the magazines reflect a diversity of tastes and are they targeted to diverse audiences? Décor that communicates a masculine culture, such as references to geeky pop culture, may signal to women and those who do not identify with these cues that they do not belong (Cheryan et al., 2009).

Research has shown that perceptions of environments are not limited to physical workspace. Websites, employment offer letters, and virtual environments have all been shown to evoke similar appraisals of belonging, potential threat, and person-organization fit to that of physical environmental cues (Ng and Burke, 2005; Braddy et al., 2006; Cheryan et al., 2011). The design and content of websites, language used in various materials, and presence of stereotypes in virtual settings all have the potential to signal to diverse applicants and employees that they do not belong (Walker et al., 2012). If organizations portray a particular culture through virtual or nontraditional avenues, and that culture could be considered threatening to women, such as one that values taking risks or that is highly competitive, the favorability of the organization from a woman’s perspective could be negatively affected. Conversely, if an organization is able to communicate an appreciation and acceptance of diversity, such as including a demographic variety in their testimonials, images, and recruiters, women’s and racial minorities’ perceptions of the organization could be bolstered (Braddy et al., 2006).

Organizational research has also shown that a stereotype-affirming environment leads members of stereotyped groups to question their belonging to that workgroup (Elsbach, 2003). Women in technology perceived greater threat when working in environments that they felt were masculine in nature. When in environments that are subtly (or not so subtly) favorable for men, it may induce women to feel that they are infiltrating a “boy’s club,” and that they must accept the existing social norms. Physical markers within an environment include things such as masculine wall colors, breakroom paraphernalia such as calendars or refrigerator magnets, or a norm of vulgar language. Making the physical environment, particularly common areas such as the breakroom and lobby, more gender neutral will help to dispel the feeling that the organization favors one gender group over the other.

In sum, employers should scrutinize physical and virtual workplace environments and messages to ensure that these cues are communicating the intended message that all employees are valued and belong.

#### Valuing Diversity Among Employees

A more pervasive environmental cue is lack of racial, ethnic, age, and gender diversity among employees. Being a numeric minority in an evaluative context such as the workplace is sufficient to trigger stereotype threat (Inzlicht and Ben-Zeev, 2000; Murphy et al., 2007). For example, women college students viewed one of two videos depicting a science conference. Those viewing the video in which women were underrepresented 3:1 were less interested in attending the conference, anticipated feeling a lack of belonging at the conference, and showed a cardiovascular threat response to watching the video compared to women who watched a gender balanced video (Murphy et al., 2007).

Research on solo status documents the negative effects of being the only or one of few members of a racial or gender group in the workplace (Saenz and Lord, 1989; Sekaquaptewa and Thompson, 2003). Numeric minorities can feel pressure to positively represent their group and engage in counter-stereotypic behavior (Saenz and Lord, 1989; Sekaquaptewa and Thompson, 2003); however, members of majority groups often attribute minority group members’ behaviors as confirming a negative group stereotype (Sekaquaptewa and Thompson, 2003). A non-diverse workforce can elicit mistrust and less commitment from minority employees (Roberson et al., 2003; Purdie-Vaughns et al., 2008).

Another reason a non-diverse workforce is problematic is there are fewer ingroup members to serve as role models for members of minority groups. Having a same-race or same-gender role model is beneficial for employees’ achievement and motivation in the domain (Dasgupta and Asgari, 2004; McIntyre et al., 2011). If ingroup role models are not available, merely presenting members of underrepresented groups with stories of successful minority role models is effective in reducing stereotype threat (von Hippel et al., 2010).

Although diversifying an organization’s workforce is the ideal solution, this may not be feasible in the short-term, particularly for smaller organizations. A possible remedy for lack of a diverse workforce is the organization’s diversity philosophy or mission. Although the organization may not have a very diverse body of employees, this does not prevent the organization from communicating its value of diversity to current and prospective employees. Research has investigated three types of diversity philosophies and their effects on minority and majority group’s perceptions of the organization, including color-blind, multicultural, and all-inclusive multicultural (Plaut et al., 2009). Although a color-blind policy indicating race does not affect performance or evaluations and employees are valued for their work ethic seems positive, this widely endorsed policy is viewed as exclusionary by minorities (Plaut et al., 2009). Often a color-blind approach results in valuing a majority perspective by ignoring important group differences and overemphasizing similarities (Ryan et al., 2007), which can in turn trigger stereotype threat (Plaut et al., 2009). In contrast, a multicultural philosophy values differences and recognizes that diversity has positive effects in organizations (Ely and Thomas, 2001). Minority groups report feeling more welcome when organizations have multicultural policies (Bonilla-Silva, 2006); however, majority groups have reported feeling excluded (Thomas, 2008). More recent research suggests an all-inclusive multicultural approach is most effective. This approach recognizes and values contributions from all groups, majority and minority, and all employees report feeling included with this philosophy (Plaut et al., 2011).

Organizational behaviors that communicate the adoption of a multicultural philosophy are often based on awareness and sensitivity. For example, the creation of a specific position responsible for managing diversity issues can better equip the organization to address diversity-related concerns. Diverse employees who are potential candidates for promotion could be identified and targeted in the promotion process. Turnover rates for diverse employees could be specifically analyzed and interpreted. Organizations can implement training with all employee ranks that stresses the value of a diverse workforce (Blanchard, 1989; Konrad and Linnehan, 1995). There are numerous strategies that organizations can undertake. Research has shown that the adoption of multicultural practices such as these leads to attracting and retaining highly qualified diverse applicants (Ng and Burke, 2005; Brenner et al., 2010), the subsequent hiring of more qualified diverse applicants (Holzer and Neumark, 2000), and greater organizational commitment among diverse employees (Hopkins et al., 2001). Conversely, if applicants perceive the organization is not welcoming of racial and ethnic diversity, they may be less likely to pursue employment with that organization (Purdie-Vaughns et al., 2008).

#### Wise Feedback and Organizational Mindset

As discussed previously, a negative consequence of stereotype threat is discounting feedback (Roberson et al., 2003), which hinders employee’s professional development and performance in the organization. Members of minority groups are particularly likely to mistrust feedback when it is in interracial or intergender contexts (Cohen et al., 1999; Cohen and Steele, 2002). A negative consequence for those giving critical feedback is the feedback withholding bias (Harber, 1998). Because giving and receiving critical feedback is important for individuals’ and organizations’ performance, employers should be trained in how to give “wise feedback” (Yeager et al., 2013). Wise feedback has the goal of clarity, to remove ambiguity regarding the motive for the feedback so that members of minority groups do not attribute negative feedback to racial or gender bias. In this approach, the supervisor communicates to the employee that he or she has high standards for the employee’s performance but that he or she believes the employee can live up to those standards. When framed in this manner, the purpose of the feedback is to help the employee meet the high standards. Field studies show that minority students given wise feedback showed more motivation to improve (Cohen et al., 1999) and were more likely to resubmit their graded work after receiving feedback (Yeager et al., 2013).

The role of communicating high standards in wise feedback is also reflective of organization mindsets. Research on entity and incremental views of intelligence (Dweck, 2006) has documented that how educators and employers, communicate their beliefs about intelligence and performance affects students’ and potential employees’ motivation and performance (Murphy and Dweck, 2010). An entity or fixed mindset reflects beliefs that intelligence is something humans are born with and that the capacity to increase intelligence occurs within innate boundaries. This mindset promotes viewing mistakes and challenges as evidence of low intelligence. In contrast, an incremental or malleable view of intelligence suggests that intelligence is a result of learning and hard work and that anyone can increase their intelligence. In this mindset, mistakes are viewed as an important part of the learning process. Research with adolescents (Paunesku et al., 2015), girls (Good et al., 2003), and racial minorities (Aronson et al., 2002) struggling with math shows that incremental mindsets predict learning and achievement. Recent work has documented that organizations perceived to have fixed mindsets elicited more stereotype threat among women (Emerson and Murphy, 2015). Organizations perceived to have a growth (incremental) mindset did not elicit threat and women reported greater trust and commitment to the organization and had higher performance (Emerson and Murphy, 2015).

In sum, supervisors should be trained in giving wise feedback. Organizations should communicate to current and prospective employees the value placed on motivation, hard work, and effort. New hires are selected in part for their competencies, thus emphasis on effort will keep employees motivated to perform well and may reduce or eliminate stereotype threat (Murphy and Dweck, 2010; Emerson and Murphy, 2015).

### Individual and Psychological Focused Interventions

#### Reattribution training

One way that employers can empower employees to avoid experiencing stereotype threat is through reattribution training, or attribution retraining (Walton and Cohen, 2007). When facing challenges common in the workplace, employees who attribute hardships to temporary, external factors are more likely to excel in the face of failure than employees who attribute setbacks to internal factors such as ability (Weiner, 1985). Research has shown that providing alternative explanations for the perceived difficulty of a task can allow individuals the opportunity to attribute that difficultly to something other than their stereotyped group membership (Wilson et al., 2002). Providing alternative explanations may help to alleviate some of the anxiety caused by stereotype threat because it buffers self-esteem from negative self-evaluation.

Research shows that reattribution training can be effective when inadequate instructions or guidelines are offered (Menec et al., 1994), employees lack practice or experience on a given task (Brown and Josephs, 1999), and the work needs to be carried out in an irregular context (Stone et al., 1999). These alternative explanations for poorer performance reflect external and less controllable circumstances, thus group membership is no longer the only plausible explanation for shortcomings in performance. The individual can now partially attribute performance to factors not associated with self-esteem.

To illustrate this technique, consider the following scenario. During the onboarding process, employers can share stories with new employees about others’ experiences when first joining company. For example, highlighting cases where individuals first felt like an outsider, but then developed a sense of community after joining an organization-related club. When a new trainee experiences difficulty learning a new job skill, the trainer can emphasize that other new employees experienced initial trouble but mastered the skill after practice, which will diffuse the negativity of the setback. However, attribution retraining is only successful when the employee is provided with the opportunity to grow and learn from their mistakes (Menec et al., 1994). Employers who wish to implement this intervention should consider the training opportunities available to new and current employees and expand resources as necessary to support development opportunities.

Attribution retraining must not be confused with simply providing plausible excuses for employees or lying to employees about why they may have failed. Additionally, attribution retraining should not give employees a guilt-free outlet for regular underperformance. Rather, the goal of attribution retraining should be to *remind* employees of any existing difficult circumstances which may be stalling performance, *not* create them (Roberson and Kulik, 2007). Therefore, managers ought to utilize this strategy only when the following criteria are met: (1) when a stereotyped employee is presumably struggling due to stereotype threat; (2) when actual difficult circumstances may be preventing employees from succeeding; and (3) when underperformance is understandable and not crucial to typical job performance. Meeting these criteria will insure that attribution retraining is targeted at combating stereotype threat among truly capable employees. Although attribution retraining will not target the source of stereotype threat, it may provide additional resources to employees who are having trouble coping with it.

#### Reframing the Task

One way in which stereotype threat can be actively removed from an evaluative performance situation is by simply reframing the task—that is, by using a description that does not evoke negative stereotypes about a social group. Although diagnostic exams and workplace evaluations activate stereotype threat implicitly, explicitly describing an exam or evaluation as non-diagnostic (for example, of intelligence) is enough to eliminate the effects of stereotype threat (Steele and Aronson, 1995). However, this method does not seem practical in diagnostic exams, such as standardized tests, that are meant to measure an individual’s academic performance. Research has also found that stereotype threat can be eliminated by explicitly stating that exams show no difference in performance based on stereotypes. For example, describing a math exam as gender-fair can be enough to dramatically increase women’s math performance (Spencer et al., 1999; Quinn and Spencer, 2001). This method is quite practical because simply stating the gender and cultural fairness of an exam before it is administered can easily reduce stereotype threat effects. In a workplace setting, describing evaluations as objective or fair may alleviate stereotype threat (Kray and Shirako, 2012). That is, if an evaluation is conducted by more than one supervisor and focuses on behaviors and quantitative metrics of performance, evaluations may be less biased and may not evoke threat (Austin and Villanova, 1992; Bommer et al., 1995). Employers should evaluate testing or evaluation procedures to make sure the fairness of the metric is communicated to employees.

#### Values-Affirmation

An intervention that can reduce stereotype threat and improve performance is values-affirmation (Sherman and Cohen, 2006; Sherman and Hartson, 2011). The intervention is based on self-affirmation theory, which states that affirming an aspect of the self that is valued and unrelated to a particular threat can buffer self-esteem and alleviate the threat (Sherman and Cohen, 2006). Value-affirmation interventions have been implemented in school settings, typically having students write for 15–20 min about things that they value and why, often including this as a regular writing assignment throughout the academic term. This helps to put students’ troubles in the broader context of their values and sources of support. This brief, low-cost intervention has shown to improve minority students’ GPA even 3 years later (Cohen et al., 2009; Sherman et al., 2013). It also has reduced stereotype threat and increased sense of belonging among minorities (Cohen et al., 2009; Sherman et al., 2013) and women in the sciences (Walton et al., 2015a). Research suggests the key mechanism for values-affirmation interventions is to have participants write about social belonging (Shnabel et al., 2013).

Recent research has applied values-affirmation interventions in the workplace and found improved performance and retention (Cable et al., 2013). Cable et al. (2013) encouraged employees of a large international organization to express their “best selves,” in that they encouraged their employees not to censor or withhold their input or perspectives. This communicated to the employees that all inputs were valued and important, and resulted in decreased experiences of stereotype threat among employees. Wiesenfeld et al. (1999) simulated an organizational layoff, in which a confederate was unfairly excused from further participation in the experiment. Results indicated that witnessing the unfair treatment, which is theorized to threaten self-integrity, inhibited performance on a subsequent task. Conversely, when the layoff was perceived as fair, participants were less likely to report self-consciousness as opposed to the unfair condition. In other words, when affected employees perceive a threat to their self-esteem, they alter how they evaluate themselves and exhibit performance detriments.

Organizations can implement brief values-affirmation interventions by providing employees with opportunities to express their values and things important in their non-work life that may boost their sense of belonging to the organization. For example, opening a business meeting by asking for announcements about recent life events such as birthdays, births, weddings, graduations, and other such positive activities highlights that organizations care about the whole person and reminds employees of the broader spectrum of their values besides their contribution to the workplace (see Lepper and Woolverton, 2002). Sharing such personal stories will likely improve interpersonal relationships among employees and with supervisors, thus improving sense of belonging to the organization (Kray and Shirako, 2012).

#### Utility-Value Interventions

Harackiewicz et al. (2015) find one reason for underachievement in academic environments is that students may not value their coursework or feel engaged in the learning process (Harackiewicz et al., 2015). Utility-value interventions aim to increase value and engagement in coursework and can combat the tendency to discount and devalue academics among students who experience stereotype threat. To be effective, such interventions must help participants value the task and believe that they can succeed at the task. Finding utility-value in the task means that individuals see the importance and usefulness of the task to accomplish their goals, both in the immediate situations and in their lives.

Harackiewicz et al. (2015) conducted an academic intervention to increase utility-value in science students by having them complete a short writing assignment in which they explained how the material they were learning (math or science) was relevant to their lives and career goals. The intervention increased perceptions of utility-value and interest, especially for students who were low in expected or actual classroom performance. Views of utility-value mediated the relationship between interests in the domain and academic performance in the domain. This intervention has been effectively implemented with first generation college students, women in biology, and racial minority students, resulting in higher end-of-semester grades. Other research finds that perception of utility-values in coursework is positively correlated with hard work, interest, and performance (Harackiewicz et al., 2008; Hulleman et al., 2008).

To our knowledge, utility-value interventions have not been implemented in the workplace. However, like values-affirmation interventions, many field studies are first conducted in education settings and later applied to organizational settings. A utility-value intervention would be useful for organizations when employees show lower motivation or interest in their work, particularly if they are performing poorly in a challenging domain like science, technology, engineering, and mathematics (STEM). For example, when learning a difficult task employees can be asked to think about how the new learning will help them accomplish their work goals, but also how it is relevant to life outside of work. However, it is important that utility-value interventions are employee-generated. That is, having supervisors tell employees that a new task is valuable is not effective and may backfire, leading to lower employee performance on the task and less interest (see Canning and Harackiewicz, 2015). A combination of direct communication about the task utility and allowing employees to self-generate the value and utility of the task is most effective. For employees lower in confidence in the task, it is more effective to apply the utility and value of the task to everyday life situations rather than to the work domain (Canning and Harackiewicz, 2015).

#### Belonging Interventions

When women and racial minorities are underrepresented in the workplace, they may experience belonging uncertainty (Walton and Cohen, 2007). When facing challenges and setbacks, members of underrepresented groups can interpret struggles as a sign that “people like me don’t belong here” (Walton and Cohen, 2007) and may feel that they alone are experiencing struggles. Belonging interventions share stories with underrepresented groups to dispel the belief that they alone feel isolated or that their difficulties are unique to their gender or racial group (Walton et al., 2015b). In academic field settings, college freshmen were given information that most college freshmen struggle with their sense of belonging in the beginning of college but that this uncertainly subsides and they develop a sense of belonging. Further, students were told that feeling a lack of belonging is experienced by all college students’ regardless of their race or gender. Compared to a control group, students who received the belonging intervention had higher GPAs throughout the entire duration of their college years (Walton and Cohen, 2011). Like reattribution training, the belonging intervention shaped the way college students interpreted their college experiences.

A naturalistic study conducted with science faculty members at a large university found evidence for belonging uncertainty (Holleran et al., 2011). Interactions among male and female faculty members were monitored for content and participants were asked to rate the competencies of those with whom they interacted. Results indicated that men were much less likely to engage in conversation regarding research with women compared to men, and when such conversations were carried out, women were generally regarded as less competent. No such competence contrasts were present for men. This imbalanced treatment appeared to evoke disengagement among women, such that inequity in socialization prompted a feeling of not belonging to the rest of the workgroup. This mirrors much of the belongingness literature regarding stereotype threat, in that performance and engagement tend to suffer for individuals who are not viewed as belonging to the group (Holleran et al., 2011).

#### Communal Goal Affordances and Interdependence

Two additional areas related to stereotype threat are closely tied to sense of belonging in university or the workplace and personal values. Research on communal goal affordances finds that women may be underrepresented in many male-dominated fields (e.g., STEM) because they do not believe these careers can meet their goals of nurturing and helping others (Diekman et al., 2010). A distinct but related concept is valuing interdependence, that underrepresented students, and by extension employees, may not see Western organizational values of independence as congruent with their values of cultural interdependence (Stephens et al., 2012b). This section reviews research and interventions on communal goal affordances, and then interdependence and cultural mismatch.

Current research suggests that women and racial minorities may experience stereotype threat in male- and majority race-dominated domains and avoid STEM disciplines because they do not see their personal life goals and cultural values as congruent with the expected quality of life of a STEM student, scientist, or engineer (Diekman et al., 2010; Smith et al., 2014, 2015; Thoman et al., 2015). Many women and racial minorities have communal goals, or an orientation to nurture others, and are more likely to endorse communal goals then men and Caucasians (Diekman et al., 2010; Smith et al., 2014; Thoman et al., 2015). Societal stereotypes of STEM disciplines suggest that scientists, mathematicians, and engineers are typically male, work in isolation in a laboratory, value competitiveness, and have little time for family (Barbercheck, 2001). Stereotypes of scientists make STEM unappealing fields of study or work for many women (Cheryan et al., 2009; Cheryan, 2012) and racial minorities, particularly those with communal goals (Diekman et al., 2010; Smith et al., 2014; Thoman et al., 2015).

One line of research examined stereotype threat through the lens of communal goals and utility-values (discussed in the previous section). Smith et al. (2015) found experiences of stereotype threat were negatively related to college women’s science identity, but this relationship was mediated by (lower) perceptions of the communal utility-value of science. Particularly among women in male-dominated majors (e.g., physics) compared to female-dominated STEM majors (e.g., biology), perceiving a career in science as less useful in reaching one’s goals to help others was related to greater experiences of stereotype threat and lower science identity (Smith et al., 2015).

An intervention with science students combined a utility-value intervention with a communal goal intervention (Brown et al., 2015). The culture of science emphasizes agentic values, which can deter women and minorities from pursuing STEM education and careers. Brown et al. (2015) found an intervention emphasizing the communal utility-value of science education, particularly addressing the desire to help others, increased students’ motivation to succeed in science.

The communal goals literature has implications for organizations in STEM fields that want to recruit a diverse workforce and support them in the workplace. It is important for organizations to communicate valuing communal goals and providing employees with opportunities to conduct work that will help the community. As with diversity policies, this can be accomplish through websites, brochures, and job descriptions. Many companies already have such opportunities in place, and contribute to local communities as part of public relations efforts. Employers should know that women, particularity in male-dominated occupations, may perceive greater fit with the organization, and therefore greater job satisfaction and performance (Spanjol et al., 2014; Svyantek et al., 2015), if they having the opportunities to reach their communal goals.

A related value that can be undermined in academic and workplace settings, and decrease sense of belonging in organizations is interdependence. Research finds that low-income, first generation college students, and racial minorities are more likely to take an interdependent worldview, compared to an independent worldview, than middle class majorities (Stephens et al., 2012a). Consistent with US culture’s emphasis on independence and agency (Markus and Kitayama, 2003), institutions of higher education promote an independent worldview (Stephens et al., 2012a). Underrepresented students may perceive a cultural mismatch and lack of fit with US universities, which predicts lower sense of belonging and academic performance (Stephens et al., 2012a).

To address this cultural mismatch in higher education, Stephens et al. (2012a) implemented a brief intervention to reframe universities’ values as fostering interdependence and tested the effects on first generation college students’ performance. During orientation, new students were randomly assigned a welcome letter from the University president that described the university’s promotion of independent or interdependent learning norms. First generation college students who received the interdependent letter had higher performance on an academic task. Further, the type of letter received affected first generation college students’ perceptions of task difficulty, linking a cultural mismatch to greater perceived difficulty and a cultural match to less difficulty. For first generation college students, those who received an interdependent letter and perceived the academic task as less difficult had better performance compared to first generation students receiving an independence letter (Stephens et al., 2012a).

The possible cultural mismatch for low-income and racial minority employees should be a concern for organizations. Organizations that promote an individualistic worldview may similarly undermine employees’ interdependence values and inadvertently alienate a segment of the workforce. The Stephens et al. (2012a) intervention could be adapted to the workplace by communicating the organizations’ value of interdependence through websites and new hire letters.

In sum, organizations can decrease underrepresented employees’ experiences of stereotype threat and increase sense of belonging by being aware of employees’ communal and interdependence goals and values. As previously stated, an all-inclusive multicultural approach is most effective for employees from all backgrounds (Plaut et al., 2011). When adopting diversity missions, philosophies, and policies, organizations can express their value of contributions from all groups, majority and minority, by including statements on how working in the organization can meet communal goals and the value placed on interdependent work.

#### Discussing Stereotype Threat

A final intervention to reduce stereotype threat in the workplace is to simply talk about it. Johns et al. (2005) explicitly told students about stereotype threat and feelings of anxiety. The researchers stated, “It’s important to keep in mind that if you are feeling anxious while taking this test, this anxiety could be the result of these negative stereotypes that are widely known in society and have nothing to do with your actual ability to do well on the tes” (Johns et al., 2005, p. 176). As a result of these instructions, women did not underperform on a math test in the stereotype threat condition. Another study found that instructing participants under stereotype threat that their anxiety may actually enhance their test performance eliminated the effect of threat (Johns et al., 2008). These studies suggest that providing people with external attributions for experiencing anxiety during evaluative performance situations may help them regulate the anxiety and reduce or eliminate stereotype threat.

Directly confronting stereotype threat can create stereotype reactance in which individuals are motivated to disprove the stereotype (Kray et al., 2001; Kray and Shirako, 2012). Kray et al. (2001, 2004) demonstrated that discussing stereotype threat created stereotype reactance and women performed better in negotiations (Kray et al., 2001, 2004) and in entrepreneurship domains (Gupta et al., 2008). Kray and Shirako (2012) suggest that organizational leaders can help reduce stereotype threat by actively managing the messages employees hear regarding what traits are necessary to perform well on tasks and ensuring that stereotypes are not activated or endorsed in the workplace.

### A Note of Caution

Researchers note that for interventions to be effective, an indirect approach should be taken (Robinson, 2010; Cohen et al., 2012). The interventions should not be advertised as a means to improve performance or well-being, as this may dampen their effects or backfire (Sherman et al., 2009). For example, employees should not be labeled as “in need” of a stereotype threat intervention, which is associated with negative consequences (Schneider et al., 1996). In the workplace, minorities who are perceived to have been hired or promoted because of affirmative action are stigmatized (Leslie et al., 2014), likewise minorities who believe they were beneficiaries of affirmative action are less satisfied and may have lower job performance (Leslie et al., 2014). Instead, interventions should be subtle, include all employees, not just minorities, and be embedded in existing workplace activities (e.g., onboarding, training, department meetings; Cohen et al., 2012).

Interventions should be focused on addressing the psychological needs and motivational processes on which they are based (Cohen et al., 2012). Interventions developed based on anecdotal evidence or intuition may backfire and create more threat (e.g., Dweck, 1999; Schneider et al., 1996). Timing of the interventions is also a factor to consider. Research is still underway to address how timing affects intervention effectiveness (Cohen et al., 2012). Interventions that focus on early stages (e.g., onboarding) serve a prevention function to intervene before the onset of stereotype threat, for example when employees are still developing their initial perceptions of the workplace. Interventions may be implemented after a problem has already been identified and can disrupt the downward spiral, for example after a merger or during a mid-quarter progress meeting (Cohen et al., 2012). It is important to note that stereotype threat interventions alone may not boost employee performance, but instead may prevent decrements in performance. Effective interventions must be coupled with opportunities for growth and resources to provide proper training for employees. That is, the interventions will not provide employees with the necessary abilities to perform well, they merely help employees reconstrue the workplace environment in ways that allow their highest potential to surface.

Finally, not all well-developed diversity policies will have the intended positive effects on diversifying the workforce and helping minority employees feel welcome. Lab based research finds that organizations with diversity policies may be seen as fair when there is objective evidence of bias (Brady et al., 2015). That is, the mere presence of a diversity policy may lead people to believe that an organization’s actual practices are fair and undermine employee’s claims of discrimination (Dover et al., 2014). Further, organizations that have received diversity award may be perceived as fair despite evidence of unfair practices (Kaiser et al., 2013). Organizations that are serious about implementing effective diversity policies *and* practices should appoint a diversity and inclusion officer with expertise in diversity science (Plaut, 2010). We, and others, argue that knowledge of employment and discrimination law is not sufficient. An expert in diversity science, and the psychology behind diversity policies and practices, is needed to fully utilize effective policies and practices to achieve diversity and inclusion in organizations (Plaut, 2010).

## Summary, Limitations, and Future Directions

In this review we have argued the recent question of scholarly debate (Kalokerinos et al., 2014), “Is stereotype threat a useful construct for organizational psychology research and practice?” reflects a research-practice gap in I/O psychology. We and others (Kray and Shirako, 2012; Kalokerinos et al., 2014) argue that stereotype threat research is highly relevant to the field of I/O psychology and should be at the forefront of research on diversity and inclusion. Throughout the review we described several field studies both within education and workplace environments. However, we recognize a dearth of studies in workplaces and this gap needs to be addressed in future research (Kray and Shirako, 2012; Kalokerinos et al., 2014).

This review provided evidence that stereotype threat affects women and racial minorities in important ways besides performance including affecting domain identification, job engagement, career aspiration, and openness to feedback. Stereotype threat is also relevant in domains beyond personnel selection including leadership, entrepreneurship, negotiations, and competitiveness. It is important to note that our review focused primarily on cognitive stereotypes and workplace behaviors beyond performance (Spencer et al., 2015). Recent research suggests that non-cognitive stereotypes have been largely ignored in the organizational stereotype threat literature (Dhanani and Wolcott, 2014). For example, the stereotype of African Americans as aggressive may affect African Americans’ workplace behaviors (e.g., withholding information or being less assertive) because of stereotype threat. This reflects a cognitive bias in the stereotype threat literature and future research should explore the role of non-cognitive stereotypes in stereotype threat in the workplace.

In this review we focused primarily on workplace behaviors other than performance, which resulted in excluding research on age-based stereotype threat and job performance (von Hippel et al., 2013; Cox, 2014; Kulik, 2014). von Hippel et al. (2013) found that older employees who experienced age-based stereotype threat reported more negative job attitudes and poorer work mental health. Negative job attitudes predicted greater intentions to resign or retire. The most common stereotypes associated with older adults are primarily cognitive or physical such as having poor memory, slower processing, reduced executive functions, and less physical speed and strength (Cuddy et al., 2005). To our knowledge, research has not examined the effects of age-based stereotype threat on non-performance outcomes such as leadership, entrepreneurship, negotiations, and competitiveness, thus literature on age-based stereotype threat was omitted. As Cox (2014) and Kulik (2014) argue, age-based stereotype threat is an understudied area and is critical for the future of organizational psychology as the workforce ages and generations intermix in the workplace. Finally, there are other types of stigmas relevant to workplace stereotype threat that were not discussed include obese employees (Carlson and Seacat, 2014) and employees with non-traditional work histories (Melloy and Liu, 2014)

We concluded the review with examples from field-tested interventions that implementing brief, low-cost workplace interventions to reduce stereotype threat is feasible. Many of the psychological processes underlying threat can be addressed in onboarding and training programs. For example, onboarding programs can implement reattribution training and belongingness interventions and a few examples were provided. Good practices in new hire training and onboarding often already reflect some of these principles (Klein and Polin, 2012).

### Where Do We Go From Here?

Although the evidence suggests that stereotype threat is highly likely to occur in workplace settings, more evidence is needed to document its occurrence (see Hall et al., 2015; von Hippel et al., 2015). In addition, some research questions remain unanswered regarding whether boundary conditions found in the lab apply in the field. As previously stated, stereotype threat does not affect all minority groups equally (Schmader et al., 2008; Logel et al., 2012) as there are many moderating variables reflecting aspects of the situation and the person. Some of the features of the situation, such as task difficulty and task diagnosticity, or the person such as high domain identification, may not be present in non-lab settings such as the workplace (Sackett and Ryan, 2012). Thus it is not clear that group identity must be high in evaluative situations with important consequences. Research needs to determine what impact the presence of absence of these variables has on stereotype threat effects in the workplace. In addition, the overemphasis on performance needs to be remedied by focusing on other outcomes important in the workplace (Kray and Shirako, 2012; Kang and Inzlicht, 2014; Spencer et al., 2015).

Two additional areas for future research that seem to be understudied concern clarifying the construct of stereotype threat (Shapiro and Neuberg, 2007; Voyles et al., 2014; Finkelstein et al., 2015) and conceptualizing measurement (Xavier et al., 2014). First, Voyles et al. (2014) argue that a similar body of literature on metastereotypes has been ignored in the stereotype literature and the stereotype threat literature includes some construct overlap with metastereotypes. Metastereotypes are people’s beliefs about what stereotypes others hold about them (Voyles et al., 2014). Therefore, metastereotypes must precede stereotype threat because stereotyped groups must believe that the perceiver holds a negative stereotype about their social group. Conceptualized this way, metastereotypes are relevant at the stereotype activation phase and stereotype threat is the reaction to the metastereotype. Future research should continue to clarify these concepts and examine the specific processes through which they operate.

Related to metastereotypes is a concern regarding how we measure self-reported experiences of stereotype threat (Xavier et al., 2014). Some of the most widely used measures seem to be measuring metastereotypes (“Some of my colleagues feel I’m not as committed because of my gender”; von Hippel et al., 2013) rather than fear about being judged with a stereotype (“I worry that if I perform poorly on this test, others will attribute my poor performance to my race”; Marx and Goff, 2005; Xavier et al., 2014). Further, Shapiro and Neuberg (2007), Shapiro (2012), Shapiro et al. (2013) have noted both construct confusion and measurement concerns in their multi-threat framework. Shapiro’s work demonstrates there are multiple forms of stereotype threat, for example threats to the self and threats to one’s group. The form of stereotype threat affects how it is measured (Shapiro, 2012) and what interventions are most appropriate (Shapiro et al., 2013).

In conclusion, research on stereotype threat is highly relevant to I/O psychology and ripe for future discoveries. What we have learned from lab and field studies is valuable for improving diversity and inclusion in organizations. Future research should continue examining the basic mechanisms and boundary conditions of stereotype threat and testing the effectiveness of interventions for the workplace.

## Author Contributions

BC conceptualized the argument and organization of the review. Each author equally contributed to the content of the review.

## Conflict of Interest Statement

The authors declare that the research was conducted in the absence of any commercial or financial relationships that could be construed as a potential conflict of interest.

## References

[B1] Acs Z. Arenius P. Hay M. Minniti M. (2005). *Global Entrepreneurship Monitor. Executive Report 2004.* Babson Park, MA: Babson College London Business School.

[B2] Anna A. L. Chandler G. N. Jansen E. Mero N. P. (2000). Women business owners in traditional and non-traditional industries. *J. Bus. Venturing* 15 279–303. 10.1016/S0883-9026(98)00012-3

[B3] Aronson J. Dee T. (2012). “Stereotype threat in the real world,” in *Stereotype Threat: Theory, Processes, and Application*, eds Inzlicht M. Schmader T. (New York, NY: Oxford), 264–279.

[B4] Aronson J. Fried C. B. Good C. (2002). Reducing the effects of stereotype threat on African American college students by shaping theories of intelligence. *J. Exp. Soc. Psychol.* 38 113–125. 10.1006/jesp.2001.1491

[B5] Ashford S. J. Northcraft G. B. (1992). Conveying more (or less) than we realize: the role of impression-management in feedback-seeking. *Organ. Behav. Hum. Decis. Process.* 53 310–334. 10.1016/0749-5978(92)90068-I

[B6] Ashford S. J. Tsui A. S. (1991). Self-regulation for managerial effectiveness: the role of active feedback seeking. *Acad. Manage. J.* 34 251–280. 10.2307/256442

[B7] Austin J. T. Villanova P. (1992). The criterion problem: 1917–1992. *J. Appl. Psychol.* 77 836–874. 10.1037/0021-9010.77.6.836

[B8] Bandura A. (1977). Self-efficacy: toward a unifying theory of behavioral change. *Psychol. Rev.* 84 191–215. 10.1037/0033-295X.84.2.191 847061

[B9] Barbercheck M. (2001). “Mixed messages: men and women in advertisements in science,” in *Women, Science, and Technology: A Reader in Feminist Science Studies*, eds Wyer M. Giesman D. Barbercheck M. Ozturk H. Wayne M. (New York, NY: Routledge Publishing), 117–132.

[B10] Blanchard F. (1989). “Effective affirmative action programs,” in *Affirmative Action in Perspective*, eds Blanchard F. A. Crosby F. J. (New York, NY: Springer-Verlag), 193–208.

[B11] Blascovich J. Mendes W. B. Seery M. D. (2004a). “Intergroup encounters and threat,” in *From Prejudice to Intergroup Emotions: Differentiated Reactions to Social Groups*, eds Mackie D. M. Smith E. R. (Hove: Psychology Press), 89–109.

[B12] Blascovich J. Seery M. D. Mugridge C. A. Norris R. K. Weisbuch M. (2004b). Predicting athletic performance from cardiovascular indexes of challenge and threat. *J. Exp. Soc. Psychol.* 40 683–688. 10.1016/j.jesp.2003.10.007

[B13] Blascovich J. Spencer S. J. Quinn D. Steele C. (2001). African Americans and high blood pressure: the role of stereotype threat. *Psychol. Sci.* 12 225–229. 10.1111/1467-9280.00340 11437305

[B14] Bommer W. H. Johnson J. L. Rich G. A. Podsakoff P. M. MacKenzie S. B. (1995). On the interchangeability of objective and subjective measures of employee performance: a meta-analysis. *Pers. Psychol.* 48 3–27. 10.1111/j.1744-6570.1995.tb01772.x

[B15] Bonilla-Silva E. (2006). *Racism without Racists: Color-Blind Racism and the Persistence of Racial Inequality in the United States*, 2nd Edn New York, NY: Rowman & Littlefield Publishers.

[B16] Braddy P. Meade A. Kroustalis C. (2006). Organizational recruitment website effects on viewers’ perceptions of organizational culture. *J. Bus. Psychol.* 20 525–543. 10.1037/a0025847

[B17] Brady L. M. Kaiser C. R. Major B. Kirby T. A. (2015). It’s fair for us: diversity structures cause women to legitimize discrimination. *J. Exp. Soc. Psychol.* 57 100–110. 10.1016/j.jesp.2014.11.010

[B18] Brenner G. Menzies T. Dionne L. Filion L. (2010). How location and ethnicity affect ethnic entrepreneurs in three Canadian cities. *Thunderbird Int. Bus. Rev.* 52 153–171. 10.1002/tie.20321

[B19] Brown E. R. Smith J. L. Thoman D. B. Allen J. M. Muragishi G. (2015). From bench to bedside: a communal utility value intervention to enhance students’ biomedical science motivation. *J. Educ. Psychol.* 107 1116–1135. 10.1037/edu0000033 26617417 PMC4657866

[B20] Brown R. P. Josephs R. A. (1999). A burden of proof: stereotype relevance and gender differences in math performance. *J. Pers. Soc. Psychol.* 76 246–257. 10.1037/0022-3514.76.2.246

[B21] Cable D. M. Gino F. Staats B. R. (2013). Breaking them in or eliciting their best? Reframing socialization around newcomers’ authentic self-expression. *Adm. Sci. Q.* 58 1–36. 10.1177/0001839213477098

[B22] Canning E. A. Harackiewicz J. M. (2015). Teach it, don’t preach it: the differential effects of directly-communicated and self-generated utility–value information. *Motiv. Sci.* 1 47–71. 10.1037/mot0000015 26495326 PMC4610746

[B23] Carlson J. H. Seacat J. D. (2014). Multiple threat: overweight/obese women in the workplace. *Ind. Organ. Psychol.* 7 469–474. 10.1111/iops.12183

[B24] Casad B. J. Merritt S. M. (2014). The importance of stereotype threat mechanisms in workplace outcomes. *Ind. Organ. Psychol.* 7 413–419. 10.1111/iops.12170

[B25] Chen E. Matthews K. A. (2003). Development of the cognitive appraisal and understanding of social events (CAUSE) videos. *Health Psychol.* 22 106–110. 10.1037/0278-6133.22.1.106 12558208

[B26] Cheryan S. (2012). Understanding the paradox in math-related fields: why do some gender gaps remain while others do not? *Sex Roles* 66 184–190. 10.1007/s11199-011-0060-z

[B27] Cheryan S. Meltzoff A. Kim S. (2011). Classrooms matter: the design of virtual classrooms influences gender disparities in computer science classes. *Comput. Educ.* 57 1825–1835. 10.1016/j.compedu.2011.02.004

[B28] Cheryan S. Plaut V. C. Davies P. G. Steele C. M. (2009). Ambient belonging: how stereotypical cues impact gender participation in computer science. *J. Pers. Soc. Psychol.* 97 1045–1060. 10.1037/a0016239 19968418

[B29] Cohen G. L. Garcia J. (2008). Identity, belonging, and achievement: a model, interventions, implications. *Curr. Dir. Psychol. Sci.* 17 365–369. 10.1111/j.1467-8721.2008.00607.x

[B30] Cohen G. L. Garcia J. Apfel N. Master A. (2006). Reducing the racial achievement gap: a social-psychological intervention. *Science* 313 1307–1310. 10.1126/science.1128317 16946074

[B31] Cohen G. L. Garcia J. Purdie-Vaughns V. Apfel N. Brzustoski P. (2009). Recursive processes in self-affirmation: intervening to close the minority achievement gap. *Science* 324 400–403. 10.1126/science.1170769 19372432

[B32] Cohen G. L. Purdie-Vaughns V. Garcia J. (2012). “An identity threat perspective on intervention,” in *Stereotype Threat: Theory, Process, and Applications*, eds Inzlicht M. Schmader T. (New York, NY: Oxford University Press), 280–296.

[B33] Cohen G. L. Steele C. M. (2002). “A barrier of mistrust: how negative stereotypes affect cross-race mentoring,” in *Improving Academic Achievement: Impact of Psychological Factors on Education*, ed. Aronson J. (San Diego, CA: Academic Press), 303–327.

[B34] Cohen G. L. Steele C. M. Ross L. D. (1999). The mentor’s dilemma: providing critical feedback across the racial divide. *Pers. Soc. Psychol. Bull.* 25 1302–1318. 10.1177/0146167299258011

[B35] Cox C. B. (2014). Miles to go: continuing to explore the effects of stereotype threat on older trainees. *Ind. Organ. Psychol.* 7 466–468. 10.1111/iops.12182

[B36] Crocker J. Major B. (1989). Social stigma and self-esteem: the self-protective properties of stigma. *Psychol. Rev.* 96 608–630. 10.1037/0033-295X.96.4.608

[B37] Crocker J. Major B. Steele C. (1998). “Social stigma,” in *The Handbook of Social Psychology* Vol. 2 eds Gilbert D. T. Fiske S. T. Lindzey G. (Boston, MA: McGraw-Hill), 504–553.

[B38] Crocker J. Voelkl K. Testa M. Major B. (1991). Social stigma: the affective consequences of attributional ambiguity. *J. Pers. Soc. Psychol.* 60 218–228. 10.1037/0022-3514.60.2.218 8421252

[B39] Croizet J. C. Després G. Gauzins M. E. Huguet P. Leyens J. P. Méot A. (2004). Stereotype threat undermines intellectual performance by triggering a disruptive mental load. *Pers. Soc. Psychol. Bull.* 30 721–731. 10.1177/0146167204263961 15155036

[B40] Cuddy A. J. Norton M. I. Fiske S. T. (2005). This old stereotype: the pervasiveness and persistence of the elderly stereotype. *J. Soc. Issues* 61 267–285. 10.1111/j.1540-4560.2005.00405.x

[B41] Cullen M. J. Hardison C. M. Sackett P. R. (2004). Using SAT-grade and ability-job performance relationships to test predictions derived from stereotype threat theory. *J. Appl. Psychol.* 89 220–230. 10.1037/0021-9010.89.2.220 15065971

[B42] Dasgupta N. Asgari S. (2004). Seeing is believing: exposure to counterstereotypic women leaders and its effect on the malleability of automatic gender stereotyping. *J. Exp. Soc. Psychol.* 40 642–658. 10.1016/j.jesp.2004.02.003

[B43] Davies P. G. Spencer S. J. Steele C. M. (2005). Clearing the air: identity safety moderates the effects of stereotype threat on women’s leadership aspirations. *J. Pers. Soc. Psychol.* 88 276–287. 10.1037/0022-3514.88.2.276 15841859

[B44] Dhanani L. Y. Wolcott A. M. (2014). The missing piece: noncognitive stereotypes and stereotype threat. *Ind. Organ. Psychol.* 7 422–424. 10.1111/iops.12172

[B45] Diekman A. B. Brown E. R. Johnston A. M. Clark E. K. (2010). Seeking congruity between goals and roles a new look at why women opt out of science, technology, engineering, and mathematics careers. *Psychol. Sci.* 21 1051–1057. 10.1177/0956797610377342 20631322

[B46] Dover T. L. Major B. Kaiser C. R. (2014). Diversity initiatives, status, and system-justifying beliefs: when and how diversity efforts de-legitimize discrimination claims. *Group Process. Intergroup Relat.* 17 485–493. 10.1177/1368430213502560

[B47] Dweck C. (2006). *Mindset: The New Psychology of Success.* New York, NY: Random House.

[B48] Dweck C. S. (1999). Caution–praise can be dangerous. *Am. Educ.* 23 4–9.

[B49] Elsbach K. D. (2003). Relating physical environment to self-categorizations: identity threat and affirmation in a non-territorial office space. *Adm. Sci. Q.* 48 622–654. 10.2307/3556639

[B50] Ely R. J. Thomas D. A. (2001). Cultural diversity at work: the effects of diversity perspectives on work group processes and outcomes. *Adm. Sci. Q.* 46 229–273. 10.2307/2667087

[B51] Emerson K. T. Murphy M. C. (2015). A company I can trust? Organizational lay theories moderate stereotype threat for women. *Pers. Soc. Psychol. Bull.* 41 295–307. 10.1177/0146167214564969 25534242

[B52] Finkelstein L. M. King E. B. Voyles E. C. (2015). Age metastereotyping and cross-age workplace interactions: a meta view of age stereotypes at work. *Work Aging Retire.* 1 26–40. 10.1093/workar/wau002

[B53] Gneezy U. Niederle M. Rustichini A. (2003). Performance in competitive environments: gender differences. *Q. J. Econ.* 118 1049–1074. 10.1162/00335530360698496

[B54] Good C. Aronson J. Inzlicht M. (2003). Improving adolescents’ standardized test performance: an intervention to reduce the effects of stereotype threat. *J. Appl. Dev. Psychol.* 24 645–662. 10.1016/j.appdev.2003.09.002

[B55] Günther C. Ekinci N. A. Schwieren C. Strobel M. (2010). Women can’t jump?—An experiment on competitive attitudes and stereotype threat. *J. Econ. Behav. Organ.* 75 395–401. 10.1016/j.jebo.2010.05.003

[B56] Gupta V. K. Bhawe N. M. (2007). The influence of proactive personality and stereotype threat on women’s entrepreneurial intentions. *J. Leadersh. Organ. Stud.* 13 73–85. 10.1177/10717919070130040901

[B57] Gupta V. K. Turban D. B. Bhawe N. M. (2008). The effect of gender stereotype activation on entrepreneurial intentions. *J. Appl. Psychol.* 93 1053–1061. 10.1037/0021-9010.93.5.1053 18808225

[B58] Hall W. M. Schmader T. Croft E. (2015). Engineering exchanges: daily social identity threat predicts burnout among female engineers. *Soc. Psychol. Pers. Sci.* 6 528–534. 10.1177/1948550615572637

[B59] Harackiewicz J. M. Canning E. A. Tibbetts Y. Priniski S. J. Hyde J. S. (2015). Closing achievement gaps with a utility-value intervention: disentangling race and social class. *J. Pers. Soc. Psychol.* 10.1037/pspp0000075 [Epub ahead of print]. PMC4853302 26524001

[B60] Harackiewicz J. M. Durik A. M. Barron K. E. Linnenbrink-Garcia L. Tauer J. M. (2008). The role of achievement goals in the development of interest: reciprocal relations between achievement goals, interest, and performance. *J. Educ. Psychol.* 100 105–122. 10.1037/0022-0663.100.1.105

[B61] Harber K. D. (1998). Feedback to minorities: evidence of a positive bias. *J. Pers. Soc. Psychol.* 74 622–628. 10.1037/0022-3514.74.3.622 9523409

[B62] Harter J. K. Schmidt F. L. Hayes T. L. (2002). Business-unit-level relationship between employee satisfaction, employee engagement, and business outcomes: a meta-analysis. *J. Appl. Psychol.* 87 268–279. 10.1037/0021-9010.87.2.268 12002955

[B63] Holleran S. E. Whitehead J. Schmader T. Mehl M. R. (2011). Talking shop and shooting the breeze: a study of workplace conversation and job disengagement among STEM faculty. *Soc. Psychol. Pers. Sci.* 2 65–71. 10.1177/1948550610379921

[B64] Holzer H. Neumark D. (2000). What does affirmative action do? *Ind. Labor Relat. Rev.* 53 240–271. 10.2307/2696075

[B65] Hopkins W. Hopkins S. Mallette P. (2001). Diversity and managerial value commitment: a test of some proposed relationships. *J. Manag. Issues* 13 288–306.

[B66] Hoyt C. L. (2005). The role of leadership efficacy and stereotype activation in women’s identification with leadership. *J. Leadersh. Organ. Stud.* 11 2–14. 10.1177/107179190501100401

[B67] Hoyt C. L. Johnson S. K. Murphy S. E. Skinnell K. H. (2010). The impact of blatant stereotype activation and group sex-composition on female leaders. *Leadersh. Q.* 21 716–732. 10.1016/j.leaqua.2010.07.003

[B68] Hulleman C. S. Durik A. M. Schweigert S. B. Harackiewicz J. M. (2008). Task values, achievement goals, and interest: an integrative analysis. *J. Educ. Psychol.* 100 398–416. 10.1037/0022-0663.100.2.398

[B69] Inzlicht M. Ben-Zeev T. (2000). A threatening intellectual environment: why females are susceptible to experiencing problem-solving deficits in the presence of males. *Psychol. Sci.* 11 365–371. 10.1111/1467-9280.00272 11228906

[B70] Inzlicht M. Good C. Levin S. van Laar C. (2006a). “How environments can threaten academic performance, self-knowledge, and sense of belonging,” in *Stigma and Group Inequality: Social Psychological Perspectives*, eds Levin S. Van Laar C. (Hove: Psychology Press).

[B71] Inzlicht M. McKay L. Aronson J. (2006b). Stigma as ego depletion how being the target of prejudice affects self-control. *Psychol. Sci.* 17 262–269. 10.1111/j.1467-9280.2006.01695.x 16507068

[B72] Inzlicht M. Kang S. K. (2010). Stereotype threat spillover: how coping with threats to social identity affects aggression, eating, decision making, and attention. *J. Pers. Soc. Psychol.* 99 467–481. 10.1037/a0018951 20649368

[B73] Inzlicht M. Schmeichel B. J. Macrae C. N. (2014). Why self-control seems (but may not be) limited. *Trends Cogn. Sci.* 18 127–133. 10.1016/j.tics.2013.12.009 24439530

[B74] Inzlicht M. Tullett A. M. Gutsell J. N. (2012). “Stereotype threat spillover: the short-and long-term effects of coping with threats to social identity,” in *Stereotype Threat: Theory, Process, and Applications*, eds Inzlicht M. Schmader T. (New York, NY: Oxford University Press), 107–123.

[B75] Inzlicht M. Tullett A. M. Legault L. Kang S. K. (2011). Lingering effects: stereotype threat hurts more than you think. *Soc. Issues Policy Rev.* 5 227–256. 10.1111/j.1751-2409.2011.01031.x

[B76] Jamieson J. P. Mendes W. B. Blackstock E. Schmader T. (2010). Turning the knots in your stomach into bows: reappraising arousal improves performance on the GRE. *J. Exp. Soc. Psychol.* 46 208–212. 10.1016/j.jesp.2009.08.015 20161454 PMC2790291

[B77] Johns M. Inzlicht M. Schmader T. (2008). Stereotype threat and executive resource depletion: examining the influence of emotion regulation. *J. Exp. Psychol. Gen.* 137 691–705. 10.1037/a0013834 18999361 PMC2976617

[B78] Johns M. Schmader T. Martens A. (2005). Knowing is half the battle teaching stereotype threat as a means of improving women’s math performance. *Psychol. Sci.* 16 175–179. 10.1111/j.0956-7976.2005.00799.x 15733195

[B79] Kaiser C. R. Major B. Jurcevic I. Dover T. L. Brady L. M. Shapiro J. R. (2013). Presumed fair: ironic effects of organizational diversity structures. *J. Pers. Soc. Psychol.* 104 504–519. 10.1037/a0030838 23163748

[B80] Kalokerinos E. K. von Hippel C. Zacher H. (2014). Is stereotype threat a useful construct for organizational psychology research and practice? *Ind. Organ. Psychol.* 7 381–402. 10.1111/iops.12167

[B81] Kang S. K. Inzlicht M. (2014). Stereotype threat spillover: why stereotype threat is more useful for organizations than it seems. *Ind. Organ. Psychol.* 7 452–456. 10.1111/iops.12179

[B82] Kenny E. J. Briner R. B. (2014). Stereotype threat and minority ethnic employees: what should our research priorities be? *Ind. Organ. Psychol.* 7 425–429. 10.1111/iops.12173

[B83] Kiefer A. Shih M. (2006). Gender differences in persistence and attributions in stereotype relevant contexts. *Sex Roles* 54 859–868. 10.1007/s11199-006-9051-x

[B84] Klein H. J. Polin B. (2012). “Are organizations on board with best practices onboarding,” in *The Oxford Handbook of Organizational Socialization*, ed. Wanberg C. (New York, NY: Oxford University Press), 267–287.

[B85] Koenig A. M. Eagly A. H. Mitchell A. A. Ristikari T. (2011). Are leader stereotypes masculine? A meta-analysis of three research paradigms. *Psychol. Bull.* 137 616–642. 10.1037/a0023557 21639606

[B86] Konrad A. Linnehan F. (1995). Formalized HRM structures: coordinating equal employment opportunity or concealing organizational practices? *Acad. Manage. J.* 38 787–820. 10.2307/256746

[B87] Kray L. J. Galinsky A. D. Thompson L. (2002). Reversing the gender gap in negotiations: an exploration of stereotype regeneration. *Organ. Behav. Hum. Decis. Process.* 87 386–410. 10.1006/obhd.2001.2979

[B88] Kray L. J. Reb J. Galinsky A. D. Thompson L. (2004). Stereotype reactance at the bargaining table: the effect of stereotype activation and power on claiming and creating value. *Pers. Soc. Psychol. Bull.* 30 399–411. 10.1177/0146167203261884 15070470

[B89] Kray L. J. Shirako A. (2012). “Stereotype threat in organizations: an examination of its scope, triggers, and possible interventions,” in *Stereotype Threat: Theory, Process, and Applications*, eds Inzlicht M. Schmader T. (New York, NY: Oxford University Press), 173–187.

[B90] Kray L. J. Thompson L. Galinsky A. (2001). Battle of the sexes: gender stereotype confirmation and reactance in negotiations. *J. Pers. Soc. Psychol.* 80 942–958. 10.1037/0022-3514.80.6.942 11414376

[B91] Kulik C. T. (2014). Spotlight on the context: how a stereotype threat framework might help organizations to attract and retain older workers. *Ind. Organ. Psychol.* 7 456–461. 10.1111/iops.12180

[B92] Lepper M. R. Woolverton M. (2002). “The wisdom of practice: lessons learned from the study of highly effective tutors,” in *Improving Academic Achievement: Contributions of Social Psychology*, ed. Aronson J. (New York, NY: Academic Press), 133–156.

[B93] Leslie L. M. Mayer D. M. Kravitz D. A. (2014). The stigma of affirmative action: a stereotyping-based theory and meta-analytic test of the consequences for performance. *Acad. Manage. J.* 57 964–989. 10.5465/amj.2011.0940

[B94] Logel C. R. Walton G. M. Spencer S. J. Peach J. Mark Z. P. (2012). Unleashing latent ability: implications of stereotype threat for college admissions. *Educ. Psychol.* 47 42–50. 10.1080/00461520.2011.611368

[B95] Major B. O’Brien L. T. (2005). The social psychology of stigma. *Annu. Rev. Psychol.* 56 393–421. 10.1146/annurev.psych.56.091103.070137 15709941

[B96] Major B. Quinton W. J. McCoy S. K. (2002). Antecedents and consequences of attributions to discrimination: theoretical and empirical advances. *Adv. Exp. Soc. Psychol.* 34 251–330. 10.1016/S0065-2601(02)80007-7

[B97] Major B. Schmader T. (1998). “Coping with stigma through psychological disengagement,” in *Prejudice: The Target’s Perspective*, eds Swim J. K. Stangor C. (San Diego, CA: Academic Press), 219–241.

[B98] Major B. Spencer S. Schmader T. Wolfe C. Crocker J. (1998). Coping with negative stereotypes about intellectual performance: the role of psychological disengagement. *Pers. Soc. Psychol. Bull.* 24 34–50. 10.1177/0146167298241003

[B99] Markus H. R. Kitayama S. (2003). Culture, self, and the reality of the social. *Psychol. Inq.* 14 277–283.

[B100] Markus H. R. Steele C. M. Steele D. M. (2000). “Color blindness as a barrier to inclusion: assimilation and nonimmigrant minorities,” in *Engaging Cultural Differences: The Multicultural Challenge in Liberal Democracies*, eds Shweder R. A. Minow M. (New York, NY: Russell Sage Foundation), 453–472.

[B101] Martens A. Johns M. Greenberg J. Schimel J. (2006). Combating stereotype threat: the effect of self-affirmation on women’s intellectual performance. *J. Exp. Soc. Psychol.* 42 236–243. 10.1016/j.jesp.2005.04.010

[B102] Marx D. M. Goff P. A. (2005). Clearing the air: the effect of experimenter race on target’s test performance and subjective experience. *Br. J. Soc. Psychol.* 44 645–657. 10.1348/014466604X17948 16368024

[B103] Marx D. M. Roman J. S. (2002). Female role models: protecting women’s math test performance. *Pers. Soc. Psychol. Bull.* 28 1183–1193. 10.1177/01461672022812004

[B104] McIntyre R. B. Paulson R. M. Taylor C. A. Morin A. L. Lord C. G. (2011). Effects of role model deservingness on overcoming performance deficits induced by stereotype threat. *Eur. J. Soc. Psychol.* 41 301–311. 10.1002/ejsp.774

[B105] Melloy R. Liu S. (2014). Nontraditional employment history: a less obvious source of stereotype threat. *Ind. Organ. Psychol.* 7 461–466. 10.1111/iops.12181

[B106] Menec V. H. Perry R. P. Struthers C. W. Schonwetter D. J. Hechter F. J. Eichholz B. L. (1994). Assisting at-risk college students with attributional retraining and effective teaching. *J. Appl. Soc. Psychol.* 24 675–701. 10.1111/j.1559-1816.1994.tb00607.x

[B107] Muraven M. Baumeister R. F. (2000). Self-regulation and depletion of limited resources: does self-control resemble a muscle? *Psychol. Bull.* 126 247–259. 10.1037/0033-2909.126.2.247 10748642

[B108] Muraven M. Tice D. M. Baumeister R. F. (1998). Self-control as a limited resource: regulatory depletion patterns. *J. Pers. Soc. Psychol.* 74 774–789. 10.1037/0022-3514.74.3.774 9523419

[B109] Murphy M. Dweck C. (2010). A culture of genius: how an organization’s lay theory shapes people’s cognition, affect, and behavior. *Pers. Soc. Psychol. Bull.* 36 283–296. 10.1177/0146167209347380 19826076

[B110] Murphy M. C. Steele C. M. Gross J. J. (2007). Signaling threat how situational cues affect women in math, science, and engineering settings. *Psychol. Sci.* 18 879–885. 10.1111/j.1467-9280.2007.01995.x 17894605

[B111] Ng E. Burke R. (2005). Person-organization fit and the war for talent: does diversity management make a difference? *Int. J. Hum. Resour. Manage.* 16 1195–1210. 10.1080/09585190500144038

[B112] Niederle M. Vesterlund L. (2010). Explaining the gender gap in math test scores: the role of competition. *J. Econ. Perspect.* 24 129–144. 10.1257/jep.24.2.129

[B113] Nussbaum A. D. Steele C. M. (2007). Situational disengagement and persistence in the face of adversity. *J. Exp. Soc. Psychol.* 43 127–134. 10.1016/j.jesp.2005.12.007

[B114] Paunesku D. Walton G. M. Romero C. Smith E. N. Yeager D. S. Dweck C. S. (2015). Mind-set interventions are a scalable treatment for academic underachievement. *Psychol. Sci.* 26 784–793. 10.1177/0956797615571017 25862544

[B115] Plaut V. C. (2010). Diversity science: why and how difference makes a difference. *Psychol. Inq.* 21 77–99. 10.1080/10478401003676501

[B116] Plaut V. C. Garnett F. G. Buffardi L. E. Sanchez-Burks J. (2011). “What about me?” Perceptions of exclusion and Whites’ reactions to multiculturalism. *J. Pers. Soc. Psychol.* 101 337–353. 10.1037/a0022832 21534702

[B117] Plaut V. C. Thomas K. M. Goren M. J. (2009). Is multiculturalism or color blindness better for minorities? *Psychol. Sci.* 20 444–446. 10.1111/j.1467-9280.2009.02318.x 19399972

[B118] Purdie-Vaughns V. Steele C. M. Davies P. G. Ditlmann R. Crosby J. R. (2008). Social identity contingencies: how diversity cues signal threat or safety for African Americans in mainstream institutions. *J. Pers. Soc. Psychol.* 94 615–630. 10.1037/0022-3514.94.4.615 18361675

[B119] Quinn D. M. Spencer S. J. (2001). The interference of stereotype threat with women’s generation of mathematical problem-solving strategies. *J. Soc. Issues* 57 55–71. 10.1111/0022-4537.00201

[B120] Roberson L. Deitch E. A. Brief A. P. Block C. J. (2003). Stereotype threat and feedback seeking in the workplace. *J. Vocat. Behav.* 62 176–188. 10.1016/S0001-8791(02)00056-8

[B121] Roberson L. Kulik C. T. (2007). Stereotype threat at work. *Acad. Manage. Perspect.* 21 24–40. 10.5465/AMP.2007.25356510

[B122] Robinson T. N. (2010). “Stealth interventions for obesity prevention and control: motivating behavior change,” in *Obesity Prevention: The Role of Brain and Society on Individual Behavior*, eds Dube L. Bechara A. Dagher A. Drewnowski A. LeBel J. James P. (New York, NY: Academic Press), 319–327.

[B123] Ryan A. M. Sackett P. R. (2013). “Stereotype threat in workplace assessments,” in *APA Handbook of Testing and Assessment in Psychology: Test Theory and Testing and Assessment in Industrial and Organizational Psychology* Vol. 1 eds Geisinger K. F. Bracken B. A. Carlson J. F. Hansen J. C. Kuncel N. R. Reise S. P. (Washington, DC: American Psychological Association), 661–673.

[B124] Ryan C. S. Hunt J. S. Weible J. A. Peterson C. R. Casas J. F. (2007). Multicultural and colorblind ideology, stereotypes, and ethnocentrism among Black and White Americans. *Group Process. Intergroup Relat.* 10 617–637. 10.1177/1368430207084105

[B125] Rydell R. J. McConnell A. R. Beilock S. L. (2009). Multiple social identities and stereotype threat: imbalance, accessibility, and working memory. *J. Pers. Soc. Psychol.* 96 949–966. 10.1037/a0014846 19379029

[B126] Sackett P. R. (2003). Stereotype threat in applied selection settings: a commentary. *Hum. Perform.* 16 295–309. 10.1207/S15327043HUP1603_6

[B127] Sackett P. R. Ryan A. M. (2012). “Concerns about generalizing stereotype threat research findings to operational high-stakes testing,” in *Stereotype Threat: Theory, Process, and Applications*, eds Inzlicht M. Schmader T. (New York, NY: Oxford University Press), 249–263.

[B128] Sackett P. R. Schmitt N. Ellingson J. E. Kabin M. B. (2001). High-stakes testing in employment, credentialing, and higher education: prospects in a post-affirmative-action world. *Am. Psychol.* 56 302–318. 10.1037/0003-066X.56.4.302 11330228

[B129] Saenz D. S. Lord C. G. (1989). Reversing roles: a cognitive strategy for undoing memory deficits associated with token status. *J. Pers. Soc. Psychol.* 56 698–708. 10.1037/0022-3514.56.5.698 2724065

[B130] Schmader T. Johns M. Forbes C. (2008). An integrated process model of stereotype threat effects on performance. *Psychol. Rev.* 115 336–356. 10.1037/0033-295X.115.2.336 18426293 PMC2570773

[B131] Schneider M. E. Major B. Luhtanen R. Crocker J. (1996). Social stigma and the potential costs of assumptive help. *Pers. Soc. Psychol. Bull.* 22 201–209. 10.1177/0146167296222009

[B132] Sekaquaptewa D. Thompson M. (2003). Solo status, stereotype threat, and performance expectancies: their effects on women’s performance. *J. Exp. Soc. Psychol.* 39 68–74. 10.1016/S0022-1031(02)00508-5

[B133] Settles I. H. (2004). When multiple identities interfere: the role of identity centrality. *Pers. Soc. Psychol. Bull.* 30 487–500. 10.1177/0146167203261885 15070477

[B134] Settles I. H. Sellers R. M. Damas A. Jr. (2002). One role or two? The function of psychological separation in role conflict. *J. Appl. Psychol.* 87 574–582. 10.1037/0021-9010.87.3.574 12090615

[B135] Shapiro J. R. (2012). “Types of threats: from stereotype threat to stereotype threats,” in *Stereotype Threat: Theory, Process, and Applications*, eds Inzlicht M. Schmader T. (New York, NY: Oxford University Press), 71–88.

[B136] Shapiro J. R. Neuberg S. L. (2007). From stereotype threat to stereotype threats: implications of a multi-threat framework for causes, moderators, mediators, consequences, and interventions. *Pers. Soc. Psychol. Rev.* 11 107–130. 10.1177/1088868306294790 18453458

[B137] Shapiro J. R. Williams A. M. Hambarchyan M. (2013). Are all interventions created equal? A multi-threat approach to tailoring stereotype threat interventions. *J. Pers. Soc. Psychol.* 104 277–288. 10.1037/a0030461 23088232 PMC3682115

[B138] Sherman D. K. Cohen G. L. (2006). The psychology of self-defense: self-affirmation theory. *Adv. Exp. Soc. Psychol.* 38 183–242. 10.1016/S0065-2601(06)38004-5

[B139] Sherman D. K. Cohen G. L. Nelson L. D. Nussbaum A. D. Bunyan D. P. Garcia J. (2009). Affirmed yet unaware: exploring the role of awareness in the process of self-affirmation. *J. Pers. Soc. Psychol.* 97 745–764. 10.1037/a0015451 19856999

[B140] Sherman D. K. Hartson K. A. (2011). “Reconciling self-protection with self-improvement,” in *Handbook of Self-Enhancement and Self-Protection*, eds Alicke M. D. Sedikides C. (New York City, NY: Guilford Press), 128–151.

[B141] Sherman D. K. Hartson K. A. Binning K. R. Purdie-Vaughns V. Garcia J. Taborsky-Barba S. (2013). Deflecting the trajectory and changing the narrative: how self-affirmation affects academic performance and motivation under identity threat. *J. Pers. Soc. Psychol.* 104 591–618. 10.1037/a0031495 23397969

[B142] Shnabel N. Purdie-Vaughns V. Cook J. E. Garcia J. Cohen G. L. (2013). Demystifying values-affirmation interventions: writing about social belonging is a key to buffering against identity threat. *Pers. Soc. Psychol. Bull.* 39 663–676. 10.1177/0146167213480816 23478675

[B143] Small D. A. Gelfand M. Babcock L. Gettman H. (2007). Who goes to the bargaining table? The influence of gender and framing on the initiation of negotiation. *J. Pers. Soc. Psychol.* 93 600–613. 10.1037/0022-3514.93.4.600 17892334

[B144] Smith J. L. Brown E. R. Thoman D. B. Deemer E. D. (2015). Losing its expected communal value: how stereotype threat undermines women’s identity as research scientists. *Soc. Psychol. Educ.* 18 443–466. 10.1007/s11218-015-9296-8

[B145] Smith J. L. Cech E. Metz A. Huntoon M. Moyer C. (2014). Giving back or giving up: native American student experiences in science and engineering. *Cultur. Divers. Ethnic Minor. Psychol.* 20 413–429. 10.1037/a0036945 25045952

[B146] Spanjol J. Tam L. Tam V. (2014). Employer–employee congruence in environmental values: an exploration of effects on job satisfaction and creativity. *J. Bus. Ethics* 130 117–130. 10.1007/s10551-014-2208-6

[B147] Spencer S. J. Logel C. Davies P. G. (2015). Stereotype threat. *Annu. Rev. Psychol.* 67 415–437. 10.1146/annurev-psych-073115-103235 26361054

[B148] Spencer S. J. Steele C. M. Quinn D. M. (1999). Stereotype threat and women’s math performance. *J. Exp. Soc. Psychol.* 35 4–28. 10.1006/jesp.1998.1373

[B149] Steele C. (1992). Race and the schooling of African-American Americans. *Atl. Mon.* 269 68–78.

[B150] Steele C. M. (1997). A threat in the air: how stereotypes shape intellectual identity and performance. *Am. Psychol.* 52 613–629. 10.1037/0003-066X.52.6.613 9174398

[B151] Steele C. M. Aronson J. (1995). Stereotype threat and the intellectual test performance of African Americans. *J. Pers. Soc. Psychol.* 69 797–811. 10.1037/0022-3514.69.5.797 7473032

[B152] Stephens N. M. Fryberg S. A. Markus H. R. Johnson C. S. Covarrubias R. (2012a). Unseen disadvantage: how American universities’ focus on independence undermines the academic performance of first-generation college students. *J. Pers. Soc. Psychol.* 102 1178–1197. 10.1037/a0027143 22390227

[B153] Stephens N. M. Townsend S. S. Markus H. R. Phillips L. T. (2012b). A cultural mismatch: independent cultural norms produce greater increases in cortisol and more negative emotions among first-generation college students. *J. Exp. Soc. Psychol.* 48 1389–1393. 10.1016/j.jesp.2012.07.008

[B154] Stone J. Lynch C. I. Sjomeling M. Darley J. M. (1999). Stereotype threat effects on black and white athletic performance. *J. Pers. Soc. Psychol.* 77 1213–1227. 10.1037/0022-3514.77.6.1213

[B155] Streets V. N. Major D. A. (2014). The limited utility of stereotype threat research in organizational settings. *Ind. Organ. Psychol.* 7 447–449. 10.1111/iops.12177

[B156] Stricker L. J. Ward W. C. (2004). Stereotype threat: inquiring about test takers’ ethnicity and gender, and standardized test performance. *J. Appl. Soc. Psychol.* 34 665–693. 10.1111/j.1559-1816.2004.tb02564.x

[B157] Svyantek D. J. Cullen K. L. Doerr A. (2015). “Person-organization fit and its implications for human resource management practices,” in *Legal and Regulatory Issues in Human Resources Management*, eds Sims R. R. Sauser W. J. Sims R. R. Sauser W. J. (Charlotte, NC: IAP Information Age Publishing), 315–340.

[B158] Thoman D. B. Brown E. R. Mason A. Z. Harmsen A. G. Smith J. L. (2015). The role of altruistic values in motivating underrepresented minority students for biomedicine. *Bioscience* 65 183–188. 10.1093/biosci/biu199 PMC4731875 26834259

[B159] Thomas K. M. (2008). *Diversity Resistance in Organizations.* New York, NY: Erlbaum-Taylor Francis.

[B160] Vick S. B. Seery M. D. Blascovich J. Weisbuch M. (2008). The effect of gender stereotype activation on challenge and threat motivational states. *J. Exp. Soc. Psychol.* 44 624–630. 10.1016/j.jesp.2007.02.007

[B161] von Hippel C. Issa M. Ma R. Stokes A. (2011a). Stereotype threat: antecedents and consequences for working women. *Eur. J. Soc. Psychol.* 41 151–161. 10.1002/ejsp.749

[B162] von Hippel C. Wiryakusuma C. Bowden J. Shochet M. (2011b). Stereotype threat and female communication styles. *Pers. Soc. Psychol. Bull.* 37 1312–1324. 10.1177/0146167211410439 21646549

[B163] von Hippel C. Kalokerinos E. K. Henry J. D. (2013). Stereotype threat among older employees: relationship with job attitudes and turnover intentions. *Psychol. Aging* 28 17–27. 10.1037/a0029825 22924658

[B164] von Hippel C. Sekaquaptewa D. McFarlane M. (2015). Stereotype threat among women in finance: negative effects on identity, workplace well-being, and recruiting. *Psychol. Women Q.* 39 405–414. 10.1177/0361684315574501

[B165] von Hippel C. Walsh A. M. Zouroudis A. (2010). Identity separation in response to stereotype threat. *Soc. Psychol. Pers. Sci.* 2 317–324. 10.1177/1948550610390391

[B166] Voyles E. Finkelstein L. King E. (2014). A tale of two theories: stereotype threat and metastereotypes. *Ind. Organ. Psychol.* 7 419–422. 10.1111/iops.12171

[B167] Walker H. J. Feild H. S. Bernerth J. B. Becton J. B. (2012). Diversity cues on recruitment websites: investigating the effects on job seekers’ information processing. *J. Appl. Psychol.* 97 214–224. 10.1037/a0025847 22004220

[B168] Walton G. M. Cohen G. L. (2007). A question of belonging: race, social fit, and achievement. *J. Pers. Soc. Psychol.* 92 82–96. 10.1037/0022-3514.92.1.82 17201544

[B169] Walton G. M. Cohen G. L. (2011). A brief social-belonging intervention improves academic and health outcomes of minority students. *Science* 331 1447–1451. 10.1126/science.1198364 21415354

[B170] Walton G. M. Logel C. Peach J. M. Spencer S. J. Zanna M. P. (2015a). Two brief interventions to mitigate a “chilly climate” transform women’s experience, relationships, and achievement in engineering. *J. Educ. Psychol.* 107 468–485. 10.1037/a0037461

[B171] Walton G. M. Murphy M. C. Ryan A. M. (2015b). Stereotype threat in organizations: implications for equity and performance. *Annu. Rev. Organ. Psychol. Organ. Behav.* 2 523–550. 10.1146/annurev-orgpsych-032414-111322

[B172] Weiner B. (1985). An attributional theory of achievement motivation and emotion. *Psychol. Rev.* 92 548–573. 10.1037/0033-295X.92.4.548 3903815

[B173] Wiesenfeld B. M. Brockner J. Martin C. (1999). A self-affirmation analysis of survivors’ reactions to unfair organizational downsizings. *J. Exp. Soc. Psychol.* 35 441–460. 10.1006/jesp.1999.1389

[B174] Wilson T. D. Damiani M. Shelton N. (2002). “Improving the academic performance of college students with brief attributional interventions,” in *Improving Academic Achievement: Impact of Psychological Factors on Education*, ed. Aronson J. (San Diego, CA: Academic Press), 89–108.

[B175] Xavier L. F. Fritzsche B. A. Sanz E. J. Smith N. A. (2014). Stereotype threat: how does it measure up? *Ind. Organ. Psychol.* 7 438–447. 10.1111/iops.12176

[B176] Yeager D. S. Paunesku D. Walton G. M. Dweck C. S. (2013). “How can we instill productive mindsets at scale? A review of the evidence and an initial R&D agenda,” in *White Paper Prepared for White House Meeting Excellence in Education: The Importance of Academic Mindsets*, Washington, DC.

